# A meta-proteomics approach to study the interspecies interactions affecting microbial biofilm development in a model community

**DOI:** 10.1038/s41598-017-16633-6

**Published:** 2017-11-28

**Authors:** Jakob Herschend, Zacharias B. V. Damholt, Andrea M. Marquard, Birte Svensson, Søren J. Sørensen, Per Hägglund, Mette Burmølle

**Affiliations:** 1 Section of Microbiology, Department of Biology, University of Copenhagen, Copenhagen, Denmark; 2 Department of Biotechnology and Biomedicine, Technical University of Denmark, Lyngby, Denmark; 3 Section for Immunology and Vaccinology, National Veterinary Institute, Technical University of Denmark, Lyngby, Denmark; 4 Department of Biomedical Sciences, University of Copenhagen, Copenhagen, Denmark

## Abstract

Microbial biofilms are omnipresent in nature and relevant to a broad spectrum of industries ranging from bioremediation and food production to biomedical applications. To date little is understood about how multi-species biofilm communities develop and function on a molecular level, due to the complexity of these biological systems. Here we apply a meta-proteomics approach to investigate the mechanisms influencing biofilm formation in a model consortium of four bacterial soil isolates; *Stenotrophomonas rhizophila*, *Xanthomonas retroflexus*, *Microbacterium oxydans* and *Paenibacillus amylolyticus*. Protein abundances in community and single species biofilms were compared to describe occurring inter-species interactions and the resulting changes in active metabolic pathways. To obtain full taxonomic resolution between closely related species and empower correct protein quantification, we developed a novel pipeline for generating reduced reference proteomes for spectral database searches. Meta-proteomics profiling indicated that community development is dependent on cooperative interactions between community members facilitating cross-feeding on specific amino acids. Opposite regulation patterns of fermentation and nitrogen pathways in *Paenibacillus amylolyticus* and *Xanthomonas retroflexus* may, however, indicate that competition for limited resources also affects community development. Overall our results demonstrate the multitude of pathways involved in biofilm formation in mixed communities.

## Introduction

Microbial biofilms are dynamic communities, where the architecture and community function is shaped by inter-species interactions, resulting in pH, oxygen and nutrient gradients. In natural and man-made environments, biofilms often host a plethora of different species, making it difficult to predict how these communities develop. This knowledge is however indispensable as microbial biofilms are highly associated with chronic infections^1^, colonization of catheters and implants^2^, crop pathogenicity^3–5^ and contamination of food products with spoilage and potential pathogenic strains^6,7^. Additionally, implementation of microbial communities for bioremediation^8^, waste-water treatment^9^, plant growth promotion^3–5^, and bio-energy^10^ provides environmentally friendly and low-cost substitutes to current technologies. To better harness beneficial biofilms and prevent harmful ones, there is a need to unravel the development and functionality of complex microbial communities. Importantly, knowledge of community development and functionality cannot be inferred by studying the single species constituents, as inter-species interactions severely alter the lifestyle of the community members^11^.

In a previous study, seven different bacterial species were isolated from Danish agricultural soil and screened in combinations of four for synergistic biofilm formation^12^. In more than half of the assembled communities inter-species interactions facilitated synergistic biofilm formation, compared to the best performing single species. One combination, composed of *Stenotrophomonas rhizophila*, *Xanthomonas retroflexus*, *Microbacterium oxydans* and *Paenibacillus amylolyticus*, showed a 4–5 fold increase in biofilm formation during co-cultivation. All species in the consortium were indispensable for the synergy and all increased in cell counts, compared to the single species biofilm^13^. A subsequent study indicated amino acid complementation as a potential driver behind the synergistic biofilm formation, as *Xanthomonas* uniquely altered its metabolism of different amino acids when co-cultured in the four-species consortium, compared to both mono and dual species biofilm^14^. Another study showed that the community synergy was not limited to batch cultivation, but could also be identified in continuous flow systems such as a drip flow reactor. The study also showed that the synergy was linked to a unique spatial localization of the four individual species in the four species biofilm^15^.

Meta-proteomics is an emerging technology within microbiology and several studies have successfully used the technique to describe microbial communities growing as model biofilms; including acid mine drainage^16,17^ and oral infection model biofilms^18,19^. The functional characterization of biofilm community by meta-proteomics can provide valuable information about the mechanisms driving community development and structure^20^, e.g. by providing information on how participating members cooperate and compete for nutrients and other resources^21,22^, and how metabolic activities are distributed between community members. For example a study applying meta-proteomics showed that elevated temperatures caused a community and genus level response in an acid mine drainage biofilm, as elevated temperatures increased the abundance of proteins involved in amino acid metabolism from members of the *Leptospirillum* group II 5-way, UBA genotypes and group III^17^. One of the challenges of studying complex microbial communities with current omics technologies is obtaining sufficient sampling depth and taxonomic resolution between related organisms to describe the community interactions and metabolic activity on a species and preferably strain level. This becomes even more problematic when community functionality is influenced by the low abundant community members^15,23,24^.

In the present study, we perform meta-proteomics profiling of a microbial consortium with the aim of elaborating on the inter-species interactions affecting its growth as a biofilm. To ensure proper taxonomical resolution we developed a novel pipeline that produces trimmed reference proteomes where peptides shared between two or more species are removed, which allowed species level resolution for protein identification and quantification. This approach enabled us to determine how participating members cooperate and compete for available nutrient resources, even among closely related species, in a microbial consortium.

## Results

### An experimental design for meta-proteome analysis of microbial biofilm consortia

**Figure 1:**
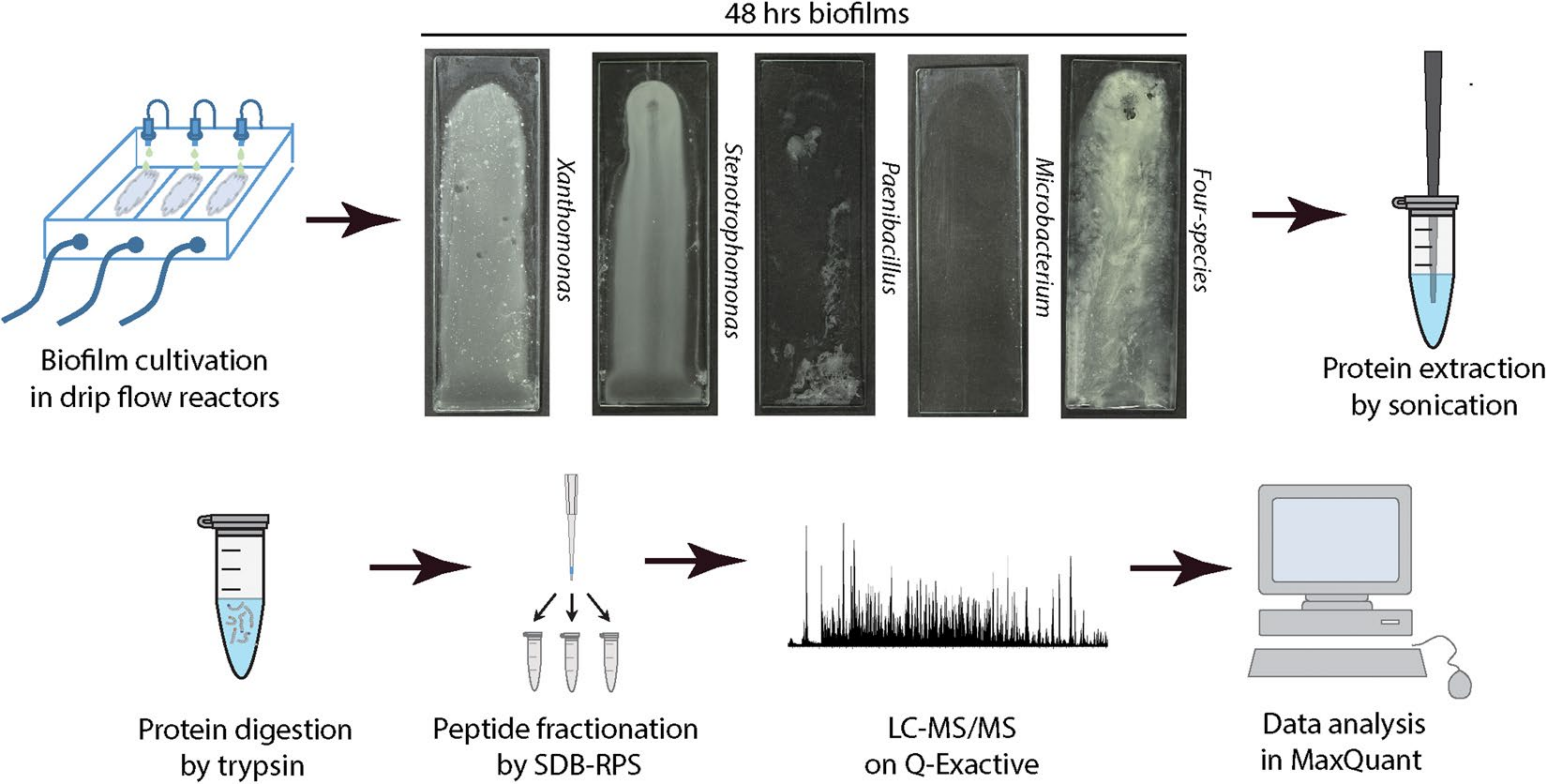
Overview of experimental design. Biofilm was cultivated in a drip flow reactor for 48 hrs and single and four-species biofilm was scraped off the glass slide. Proteins were extracted sonication, digested with trypsin and the resulting peptides were separated into three fractions using SDB-RPS filter tips. Mass spectrometric analysis was performed on a Q-Exactive and data was analyzed using MaxQuant with label free quantification.

We set out to investigate and identify inter-species interactions believed to drive community development in a previously identified synergistic biofilm consortium, including *Stenotrophomonas rhizophila (Stenotrophomonas)*, *Xanthomonas retroflexus (Xanthomonas)*, *Microbacterium oxydans (Microbacterium)* and *Paenibacillus amylolyticus (Paenibacillus)* ^13,15^. The consortium has previously been shown to interact synergistically in both a modified version of the Calgary biofilm device^25^ and in drip flow reactor (DFR) systems^15^. As the DFR enabled sufficient biomass for proteomic analysis, the consortium and single species biofilms were cultured in the DFR^26^. As the previous synergistic interactions were observed with growth in tryptic soy broth (TSB) media, TSB was also utilized in the current study to elaborate on previous observations. The DFR system allowed biofilm cultivation under low shear stress conditions in the water-air interphase (Fig. 1). Visual inspection of glass slides after two days of growth showed that the single species *Xanthomonas* and *S* *tenotrophomonas* and the four-species community yielded large amounts of biofilm as opposed to *Paenibacillus* and *Microbacterium* forming limited or no visible biofilm, respectively. No indications of dispersal were observed in the biofilms of *Xanthomonas, Stenotrophomonas* and the four-species community (e.g. reduction in biomass) and these biofilms presented continued growth, indicating mature and growing biofilms. The biofilm of the four-species consortium was visually larger, denser and had a unique texture compared to any of the single species (Fig. 1). Crystal violet staining of the glass slides confirmed that the consortium yielded larger amounts of biofilm than any of the single species (Supplementary Fig. S1). This indicated that the inter-species interactions promoting synergy were at play. Biofilms were scraped of the glass slides and proteins were extracted, proteolytically cleaved, fractionated and analysed by mass spectrometry (MS) based proteomics according to materials and methods.

### Non-overlapping reference proteomes facilitate protein identification and quantification between closely related species

Initial analysis of the MS data from the four species biofilm showed that *Paenibacillus*, *Stenotrophomonas* and *Xanthomonas* were the most dominant species in terms of identified proteins. Unfortunately, many identified proteins could not be resolved at the species level as some identified peptides from the LC-MS/MS analysis were indistinguishable between community members. In particular, the phylogenetically closely related *Stenotrophomonas* and *Xanthomonas* showed a large overlap of identified peptides (Supplementary Table S1). The occurrence of peptides shared between species hampered species level resolution of identified proteins and was believed to bias protein quantification of these proteins. To circumvent this issue, a simple pipeline was made, which generates trimmed reference proteomes. Specifically, the pipeline *in silico* cleaves each reference proteome with trypsin. The resulting peptides are compared between species and species overlapping peptides longer than seven amino acids are removed from the protein sequences. Protein sequences are re-assembled without species overlapping peptides. Trimmed reference proteomes are generated as output along with schematic representations of proteome overlap and lists of peptide trimming per protein. A schematic representation of the pipeline is shown in Supplementary Fig. S2. The minimum length of seven amino acids was used as cut-off as shorter peptides in most cases are incompatible with mass spectrometric peptide identification^27^. Supplementary Table S2 shows the number of overlapping peptides per species identified by the trimming pipeline, and Supplementary Fig. S3 shows the distribution of peptides across theoretical proteins, ordered by species. In total, 10956 peptides, corresponding to 16.1% and 17.5% of the *in silico* digested peptides from *Xanthomonas* and *Stenotrophomonas*, were shared between their reference proteomes. Other pairwise species combinations gave smaller overlaps in the range of 100 shared peptides, which for example only corresponded to 0.1% and 0.15% of *in silico* digested peptides from *Paenibacillus* and *Microbacterium*, respectively (Supplementary Table S2).

To validate the effect of trimmed reference proteomes, protein identification and quantification was assessed on a simplified part of the full data set, containing only the five biological replicates of the four-species biofilm, see data description for Supplementary Fig. S4–5. Supplementary Fig. S4a–c summarises the effect of using trimmed reference proteomes. Application of the trimmed reference proteomes significantly reduces the average number of identified proteins per sample which can’t be resolved at species level (Supplementary Fig. S4a). For the three major species in the four-species biofilm, the trimmed reference proteomes lead to reductions in the average numbers of identified proteins, (Supplementary Fig. S4b). For *Paenibacillus* and *Stenotrophomonas* this reduction was above the p-value cut off of 0.05 and hence in-significant. Combined the application of trimmed reference proteomes lead to a slight decrease in the total number of identified proteins with species level resolution for *Paenibacillus* and *Xanthomonas* with 18 and 4 proteins, respectively. However, the number of identified proteins from *Stenotrophomonas* increased with 110 proteins at species level resolution. To validate if application of the trimmed reference proteomes affected protein quantification, average protein intensities were addressed for both trimmed and non-trimmed proteins from the three most dominant species in the four-species biofilm. Application of the trimmed reference proteins significantly lowered the mean protein intensity of proteins trimmed by the pipeline for both *Xanthomonas* and *Stenotrophomonas*, see Supplementary Fig. S5a–c. Quantification of *Paenibacillus* proteins was not significantly affected by trimming of the reference proteome. Removal of shared peptides unsurprisingly allowed a more accurate protein quantification as removal of shared peptides prevented biased quantification in the four-species biofilm.

Re-analysis of the MS data with the trimmed reference proteomes removed 9324 and 7676 peptide identifications annotated to proteins from *Stenotrophomonas* and *Xanthomonas*, respectively. Supplementary Fig. S6 shows the numbers of peptides and proteins removed by the trimmed reference proteomes. Despite the substantial number of removed peptides by the trimming of *Stenotrophomonas* and *Xanthomonas*, only a small loss in the number of identified proteins was observed. Likewise, only minor differences were observed for protein identifications from *Paenibacillus* and *Microbacterium*.

To verify sufficient quality of the data obtained by the trimmed reference proteomes, the protein abundance profiles were correlated between each biological replicate for data analysed with both trimmed and standard reference proteomes (Supplementary Fig. S7–9). With the trimmed reference proteomes the single species biofilms of *Paenibacillus*, *Stenotrophomonas* and *Xanthomonas* had excellent correlations with average Pearson correlations of 0.96, 0.95 and 0.90, with slightly lower average correlations of 0.93, 0.88 and 0.82 for the four-species replicates, respectively. The correlation for *Xanthomonas* and *Stenotrophomonas* was reduced by approximately 0.02–0.1 by using the trimmed reference proteomes compared to the normal reference proteomes, which was probably due to removal of some abundant house-keeping proteins shared between the species. Application of the trimmed reference proteomes did not hamper protein normalization, and the MaxQaunt LFQ normalization algorithm was able to normalise for the difference in biomass between single- and four-species biofilm samples (Supplementary Figure S10–12). All following analyses were based on data generated with the trimmed reference proteomes.

### Meta-proteome composition and coverage

**Figure 2:**
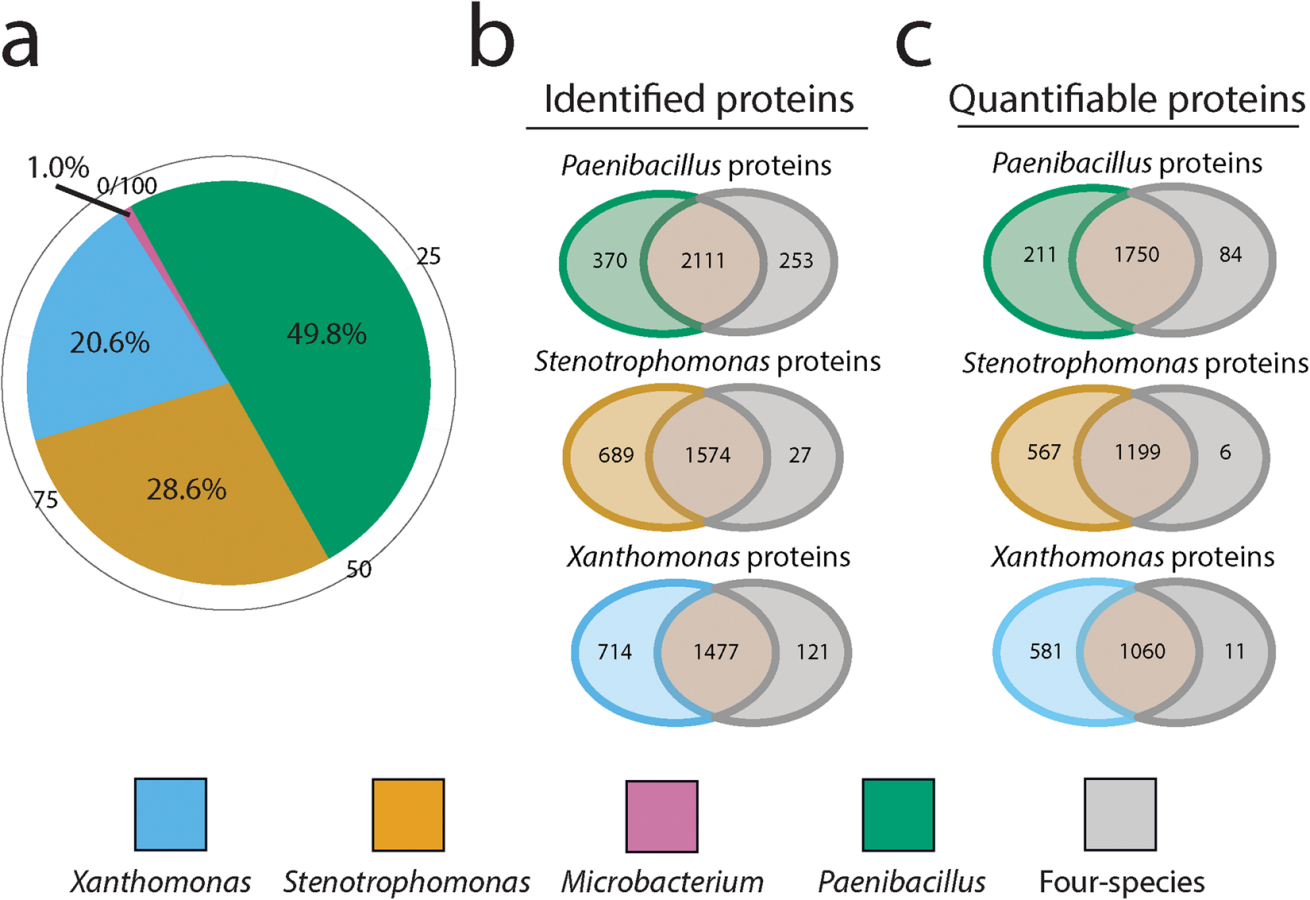
(**a**) Species composition in the four-species biofilm based on intensity of identified proteins from the four-species biofilm. (**b**) Protein overlap of identified proteins between single and four-species biofilm. More proteins were identified in the single species biofilm compared to the four-species biofilm. The majority of identified proteins were shared between single and four-species biofilm. (**c**) Protein overlap of quantifiable proteins between single and four-species biofilm. Quantifiable proteins comprised proteins occurring in at least 60% of biological replicates from each single and four-species biofilm.

The abundance of each of the four species in the biofilm consortium was estimated by comparing the distribution of protein intensity from identified proteins (Fig. 2a), and plate counts of colony forming units (CFU) (Supplementary Fig. S13). Both methods showed that all four species were present in the consortium biofilm, with *Paenibacillus* comprising approximately half, *Xanthomonas* and *Stenotrophomonas* ~21% and ~29% respectively, and *Microbacterium* ~1% (Fig. 2a). The similar species ratio from protein intensity distribution and CFU plate counts indicated that the protein extraction method was not biased towards particular species. Investigation of the dynamic range^28^ showed a span of almost 6 orders of magnitude in the multi-species biofilm (Supplementary Fig. S14), with both the most and least abundant proteins being from *Paenibacillus*. *Microbacterium* being the least abundant organism still almost spanned 4 orders of magnitude, whereas *Stenotrophomonas* and *Xanthomonas* spanned approximately 5 orders of magnitude. This indicates the increased difficulty in identifying low abundant species in a multi-species biofilm.

Protein extraction and MS analysis yielded high coverage of reference proteomes of the single species biofilms of *Stenotrophomonas* (61.6%)*, Xanthomonas* (52.8%)*, Paenibacillus* (38.6%) and of the four-species biofilm consortium (33.0%) (Table 1). Although 38.6% reference proteome coverage was obtained from the single species *Microbacterium*, the low coverage of *Microbacterium* in the four-species biofilm hampered data analysis and *Microbacterium* was therefore excluded from further data analysis. The distribution of identified proteins from the single and four-species biofilm showed that > 90% of the all the proteins identified in the four-species biofilm were also produced in the corresponding single species biofilms (Fig. 2b). Proteins occurring in at least 60% of the biological replicates from each single and four-species biofilms (Fig. 2c) were used for the quantitative meta-proteomics analysis, and are henceforth referred to as quantifiable proteins. Supplementary Table S3 shows reference proteome coverage of quantifiable proteins. Supplementary Figure S15 displays the number of identified proteins with standard reference proteomes and the overlap between single- and four-species biofilm.

**Table 1:** Overview of identified proteins and percentage of reference proteome coverage.

Samples	# Bio reps	Genome size	Protein coding genes	Identified proteins	Proteome coverage (%)
*Stenotrophomonas*	5	4.22 Mbp	3674	2263	61.6
*Xanthomonas*	5	4.68 Mbp	4149	2191	52.8
*Microbacterium*	3	3.99 Mbp	3803	1469	38.6
*Paenibacillus*	5	7.27 Mbp	6430	2481	38.6
Four-species	5	—	18056	5961	33.0

### Changes in meta-proteome profiles

**Figure 3:**
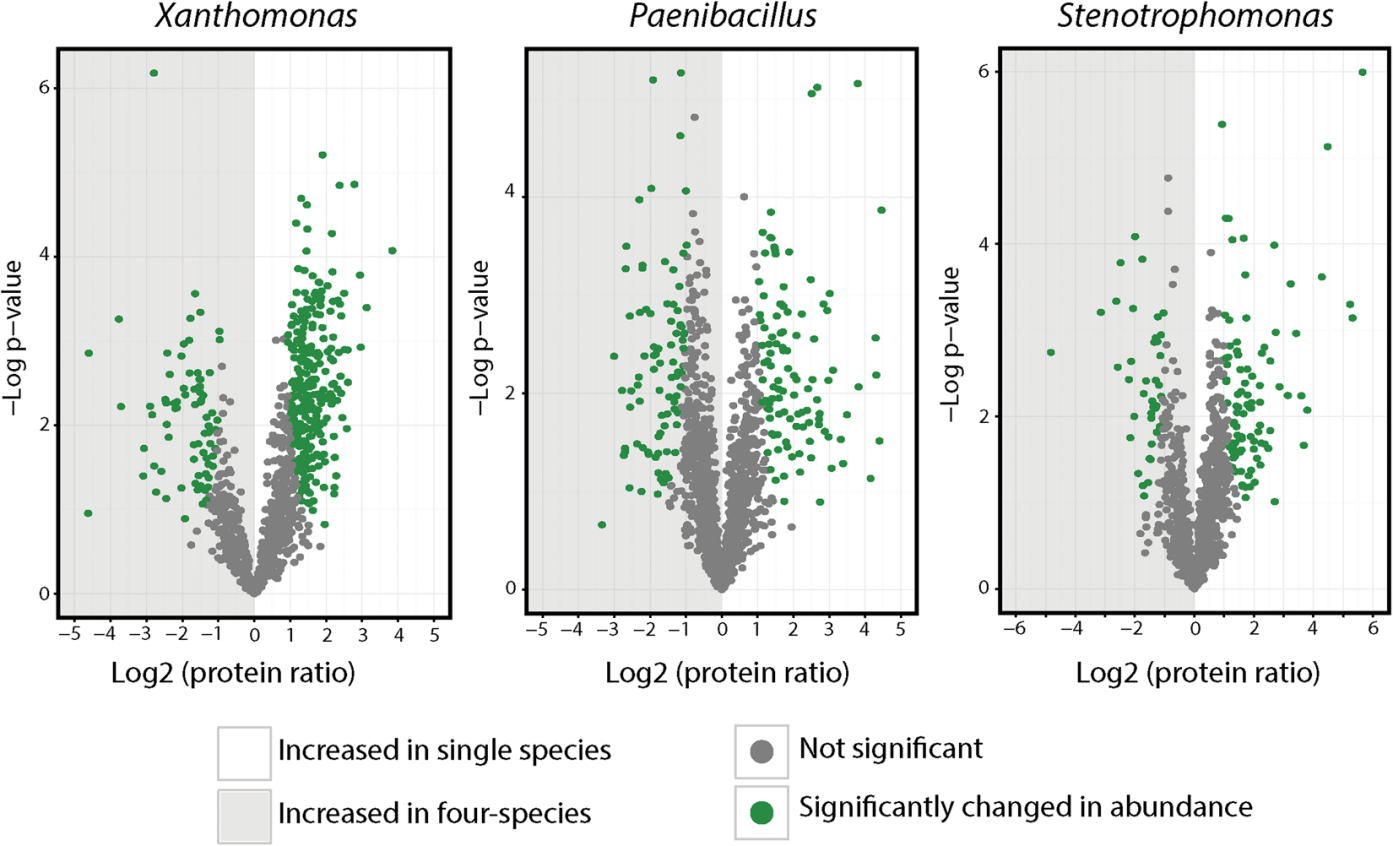
Label free quantification of proteins between five biological replicates of single- and four-species biofilm. Proteins with significant changes in abundance were identified using a modified Welsh t-test^61^ with an S0 constant of 1 and valid values in at least 60% of both single species and four-species biofilm (three out of five replicates for both conditions). Correction for multiple hypothesis testing was performed with permutation based FDR with a significance threshold of 0.05. In total, 194, 352 and 132 proteins from *Paenibacillus*, *Xanthomonas* and *Stenotrophomonas*, respectively, differed significantly between the single and four-species biofilm.

The quantitative proteomics profiles of single and four-species biofilms were compared using a Welsh t-test with a permutation based false discovery rate (FDR) of 0.05 (Fig. 3). Altogether 194, 352 and 132 proteins differed significantly in abundance between the single and the four-species biofilm for *Paenibacillus*, *Xanthomonas* and *Stenotrophomonas*, respectively, (Fig. 3). Supplementary Fig. S16 display plots with proteins having a significantly changed abundance for *Paenibacillus*, *Xanthomonas* and *Stenotrophomonas* without data normalisation by the MaxQuant LFQ normalisation algorithm.

**Figure 4:**
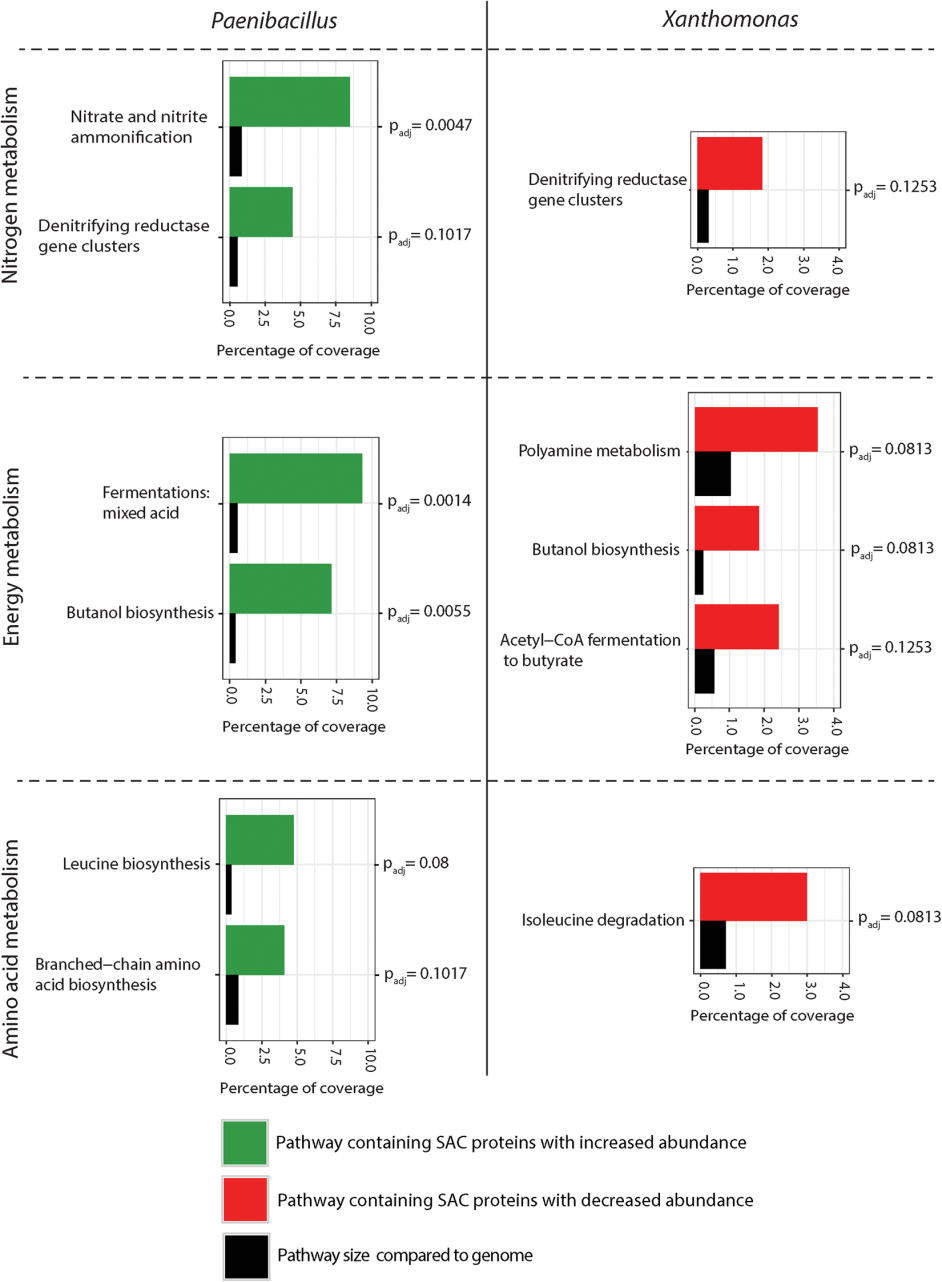
Pathways from *Xanthomonas* and *Paenibacillus* showing opposite trends in the four-species biofilm. SCA proteins were mapped with subsystems classifications from RAST. Pathways were analysed for regulation using a Fisher’s exact test. FDR adjusted p-values are presented for each pathway. Black bars display pathway size compared to the size of reference proteome, which can be grouped in pathways. Green and red bars display the ratio of SCA proteins in the pathway compared to the total amount of SCA proteins in pathways. Pathways in red further indicate decreased abundance in the four-species biofilm, and pathways in green indicate increased protein abundance in the four-species biofilm.

Proteins with significant changes in abundance (SCA proteins) were annotated to functional pathways using the subsystem features from the RAST database^29,30^. Approximately 60% of SCA proteins could be mapped to subsystem pathways. Remaining proteins were either hypothetical proteins or had known function but unknown pathway affiliation (Supplementary Table S4), stressing the need for more knowledge on protein and gene functions in microorganisms. Significantly regulated pathways were identified using Fischer’s exacts test, corrected for multiple hypothesis testing by applying a Benjamini Hochberg FDR. Significantly regulated pathways were defined as pathways overrepresented by SCA proteins, relative to the size of the annotated species proteome. The Fischer’s exacts test identified pathways containing significant amounts of SCA proteins with either decreased or increased protein abundance for *Paenibacillus* and *Xanthomonas*, when these two species participated in the four-species biofilm. Only pathways with decreased SCA proteins were identified for *Stenotrophomonas*, and these pathways were related to ribosomal subunits. Full lists of identified pathways for *Paenibacillus*, *Xanthomonas* and *Stenotrophomonas* are available in Supplementary Fig. S17-18. Comparison of regulated pathways between species revealed potential interactions between *Paenibacillus* and *Xanthomonas* regarding nitrogen, energy and amino acids metabolism, see Fig. 4.

### Nitrogen metabolism

**Figure 5:**
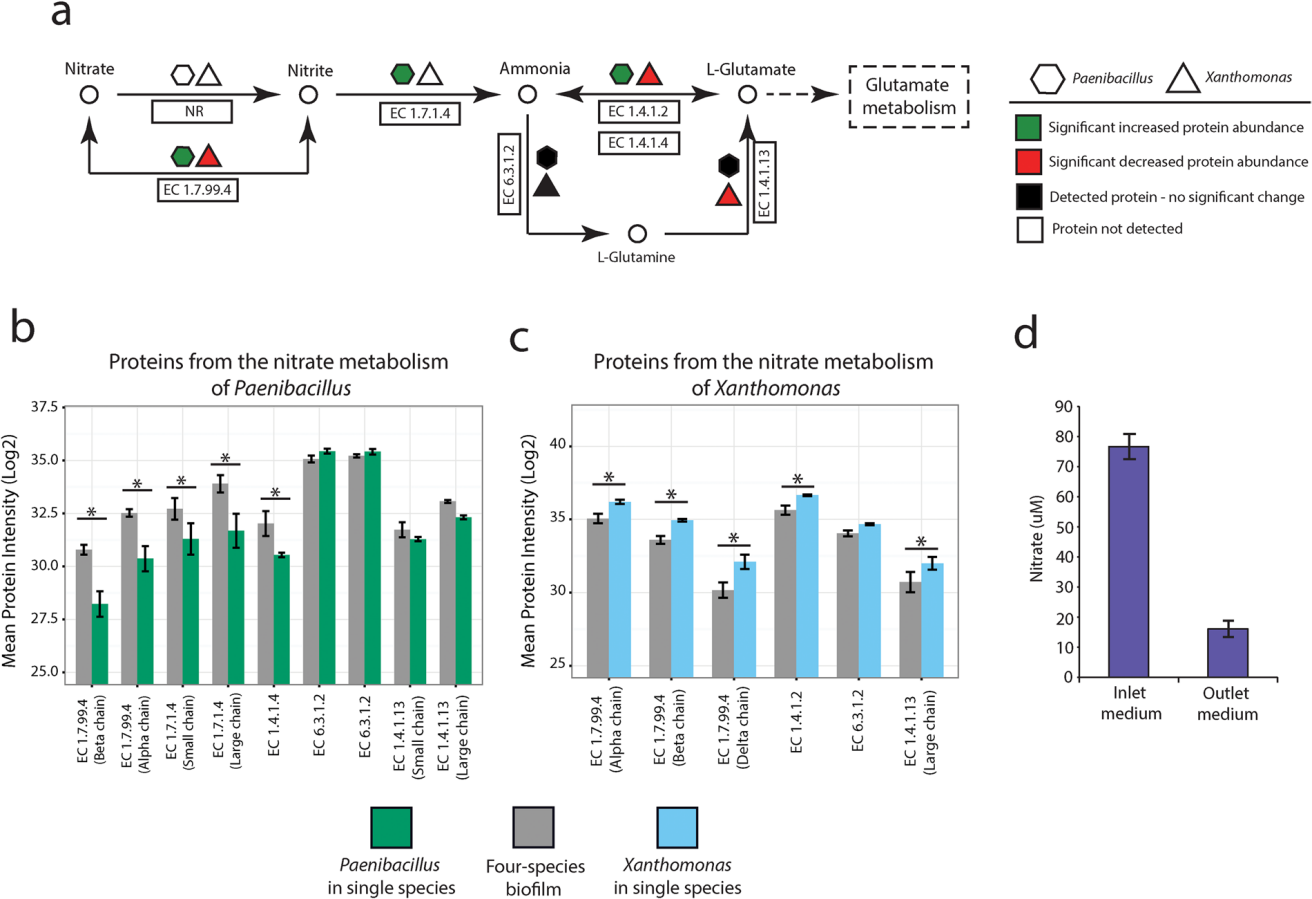
(**a**) Nitrate metabolism of *Xanthomonas* and *Paenibacillus*. Quantifiable proteins from *Xanthomonas* (triangles) and *Paenibacillus* (hexagons). Symbol color indicates if proteins are significantly increased (green) in abundance, significantly decreased (red) in abundance, not significantly changed (black), or not detected (white) in the four-species biofilm (**b**) Log2 protein intensities of quantifiable proteins from the nitrate metabolism of *Paenibaillus*. Error bars indicate confidence intervals and (*) indicates a significant difference with p < 0.05. (**c**) Log2 protein intensities of quantifiable proteins from the nitrate metabolism of *Xanthomonas*. Error bars indicate confidence intervals and (*) indicates a significant difference with p < 0.05. (**d**) Nitrate concentration in inlet and outlet medium during biofilm harvest. Error bars are standard deviations.

Pathway analysis showed increased abundance of proteins associated with nitrogen metabolism in *Paenibacillus* of the four-species biofilm compared with the corresponding single species biofilm; nitrate and nitrite ammonification (p_adj_ = 0.0047) and the denitrifying reductase gene cluster (p_adj_ = 0.1017) (Fig. 4). Both pathways contained the alpha and beta chains of the respiratory nitrate reductase (EC 1.7.99.4), which were both significantly increased in abundance. An increase in abundance was also observed for the large and small subunit of the nitrite reductase [NAD(P)H] (EC 1.7.1.4), which are part of the nitrate and nitrite ammonification pathway (Fig. 5). In contrast, in *Xanthomonas*, the proteins involved in the denitrifying reductase gene cluster (p_adj_ = 0.1253) were significantly decreased in abundance in the four-species biofilm consortium. In addition, the alpha, beta and delta chain of the respiratory nitrate reductase (EC 1.7.99.4), were all decreased in abundance in *Xanthomonas* (Fig. 5).

The opposite regulation of proteins involved in nitrogen metabolism suggests that *Xanthomonas* undergoes a metabolic shift to lower the utilization of nitrate due to the presence of *Paenibacillus* in the four-species biofilm. Simultaneously, the increased abundance of nitrate reductase indicates that *Paenibacillus* actively imports and catabolizes nitrate from the medium while producing ammonia by nitrite reductase. A further investigation of proteins related to the nitrogen metabolism showed significantly higher abundance of NAD-specific glutamate dehydrogenase (EC 1.4.1.4) from *Paenibacillus* in the four-species biofilm. The NAD-specific glutamate dehydrogenase catalyses formation of L-glutamate and nitrogen assimilation (Fig. 5a,b). In contrast, significantly decreased abundances of NAD-specific glutamate dehydrogenase (EC 1.4.1.4) and glutamate synthase [NADPH] were observed in *Xanthomonas*, suggesting a decreased assimilation of nitrogen through ammonia utilization (Fig. 5a,c). To monitor nitrate utilization dynamics, we measured the nitrate concentration in inlet and outlet medium from the drip-flow reactor, and found that approx. 80% of the nitrate was removed from the medium by the biofilm community (Fig. 5d). Additionally, ammonia measurements from inlet and outlet medium showed that the community produced ammonia (data not shown). The increase in ammonia was higher than the decrease of nitrate in the medium, and could therefore not solely be explained by reduction of nitrite by *Paenibacillus*.

### Energy Metabolism

The pathway analysis revealed also opposite trends in pathways related to fermentation in *Paenibacillus* and *Xanthomonas*. Proteins assigned to the pathways Fermentation: Mixed Acids (p_adj_ = 0.0014) and Butanol Biosynthesis (p_adj_ = 0.0055) were significantly increased in abundance in the four-species biofilm (Fig. 4). From the Fermentation: Mixed Acid pathway, the proteins pyruvate formate-lyase activating enzyme (EC 1.97.1.4), alcohol dehydrogenase (EC 1.1.1.1), pyruvate formate-lyase (EC 2.3.1.54) and acetaldehyde dehydrogenase (EC 1.2.1.10) were all more abundant in *Paenibacillus* in the four-species biofilm in comparison with the single species biofilm. Alcohol dehydrogenase, pyruvate formate-lyase and acetaldehyde dehydrogenase were all shared with the butanol biosynthesis pathway and may hence contribute to regulation of both pathways. Identification of the pyruvate formate-lyase activating enzyme (EC 1.97.1.4) supports active fermentative metabolism by *Paenibacillus*. EC 1.97.1.4 facilitates the first step in the anaerobic glucose metabolism known from *E. coli* and other facultative anaerobes^31,32^. Supporting the shift in fermentative metabolism, a shift in the central carbohydrate metabolism was also observed for *Paenibacillus*. Seven proteins from the central carbohydrate metabolism were significantly increased in abundance in the single species biofilm, whereas only one protein was significantly changed in abundance when *Paenibacillus* grew in the four-species biofilm (data not shown).

In contrast, proteins from the Butanol biosynthesis pathway (p_adj_ = 0.0813) and the Acetyl-CoA fermentation pathway (p_adj_ = 0.1253) from *Xanthomonas* were significantly decreased in abundance in the four-species biofilm. These proteins include Alcohol dehydrogenase (EC 1.1.1.1), Enoyl-CoA hydratase (EC 4.2.1.17), Acetyl-CoA acetyltransferase (EC 2.3.1.9), 3-hydroxyacyl-CoA dehydrogenase (EC 1.1.1.35) and Electron transfer flavoprotein-ubiquinone oxidoreductase (EC 1.5.5.1). Except for EC 1.1.1.35 and EC 1.5.5.1, the three other proteins were shared with the butanol biosynthesis pathway.

In support of decreased fermentation by *Xanthomonas*, several proteins from the Polyamine metabolism (p_adj_ = 0.0813) were also significantly decreased in abundance in the four-species biofilm. These proteins included a biosynthetic arginine decarboxylase (EC 4.1.1.19), a spermidine synthase (EC 2.5.1.16), N-carbamoylputrescine amidase (EC 3.5.1.53) and a putrescine ABC transporter and a protein with the dual function of an ornithine decarboxylase (EC 4.1.1.17) and an arginine decarboxylase (EC 4.1.1.19) (data not shown). Arginine decarboxylases have previously been associated with maintenance of bacterial pH homeostasis during acid stress^33,34^, which would likely occur during fermentative metabolism. Similarly, both spermidine and putrescine have been associated with acid stress^35,36^. A schematic overview of spermine formation by the arginine metabolism, with the related shifts in protein abundance from *Xanthomonas*, is presented in Supplementary Fig. S19.

### Amino acid metabolism

Compared to the single species biofilms, the following proteins were significantly less abundant in the pathway of isoleucine degradation (p_adj_ = 0.0813) when *Xanthomonas* participated in the four-species biofilm*;* 3-hydroxyacyl-CoA dehydrogenase (EC 1.1.1.35), the branched-chain acyl-CoA dehydrogenase (EC 1.3.99.12) and the Enoyl-CoA hydratase (EC 4.2.1.17). These enzymes are all uniquely involved in the degradation of valine, leucine and isoleucine. Additionally, the branched-chain amino acid aminotransferase (EC 2.6.1.42) and the leucine dehydrogenase (EC 1.4.1.9), which are all required for valine, isoleucine and leucine biosynthesis and degradation, were also significantly less abundant when *Xanthomonas* was in the four-species biofilm. As the latter enzymes catalyze both biosynthesis and degradation, their decreased abundances do not directly indicate whether these amino acids are synthesized or degraded. However, Methylcrotonyl-CoA carboxylase carboxyl transferase subunit (EC 6.4.1.4), which only catalyzes leucine degradation, was also significantly decreased in abundance when *Xanthomonas* participated in the four-species biofilm. The overall decreased abundance of enzymes involved in branched-chain amino acid metabolism suggested that *Xanthomonas* had a lower requirement for these amino acids when living in the four-species biofilm (Supplementary Fig. 19).

The pathway analysis showed that *Paenibacillus* proteins involved in biosynthesis of leucine (p_adj_ = 0.08) and branched chain amino acids (p_adj_ = 0.1171) significantly increased in abundance in *Paenibacillus* in four-species biofilm compared to single species biofilm. Within the leucine biosynthesis pathway both 3-isopropylmalate dehydrogenase (EC 1.1.1.85) and 3-isopropylmalate dehydratase large subunit (EC 4.2.1.33) were significantly increased in abundance in *Paenibacillus* in single versus four-species biofilm. This inverse regulation pattern indicates that *Xanthomonas* can cross-feed on amino acids produced by *Paenibacillus* (Supplementary Fig. S20).

## Discussion

A meta-proteomics approach was applied to unravel the inter-species interactions influencing biofilm community development from a previously identified synergistic model consortia. In agreement, with previous observations by Ren *et al*.^13^ and Liu *et al*.^15^, the four-species community had increased biofilm formation compared to the individual single species, indicating that the mechanisms driving increased biofilm formation were at play.

Ren *et al*.^13^ found that *Xanthomonas* was the best single species biofilm producer, whereas *Stenotrophomonas*, *Microbacterium* and *Paenibacillus* displayed very low biofilm yields. Both the current study and Liu *et al*.^15^ observed that *Xanthomonas* and *Stenotrophomonas* formed thick mono-species biofilms in the drip flow reactor, whereas *Microbacterium* and *Paenibacillus* were characterized as poor biofilm formers. Importantly, using a DFR Liu *et al*.^15^ observed that, at 24 hrs of biofilm growth, *Paenibacillus* significantly increased in biomass in the four-species biofilm as compared to mono and three-species biofilm combinations. It was also observed that *Microbacterium* increased in biomass, whereas *Stenotrophomonas* and *Xanthomonas* decreased in biomass in the four-species biofilm. Similarly, we observed that *Paenibacillus* became a major contributor to the four-species biofilm, comprising 57% of the total cell counts. Combined this could suggest that facilitated surface attachment plays a crucial role for *Microbacterium* and *Paenibacillus*, as these two species were unable to form thick biofilms alone and increased in occurrence in the four-species biofilm. Facilitated attachment has previously been shown for other bacterial co-cultures, for example *Listeria monocytogenes* has enhanced surface attachment on stainless steel when the surface has been pre-cultivated with *Pseudomonas putida* ^37^ and similarly, the waterborne *Escherichia coli* O157:H7 has enhanced attachment to glass when co-cultured with *Pseudomonas aeruginosa* PAO1^38^.

The meta-proteomics approach identified several mechanisms, which in concert affect community development. These mechanisms included potential competitive and facilitative interactions. *Paenibacillus* and *Xanthomonas* had opposing trends in fermentation and nitrogen metabolism, which could indicate competitive exclusion in anaerobic zones due to resource limitation. In contrast the opposing trend in branched-chain amino acid metabolism could reflect cooperative cross-feeding.

*Xanthomonas* decreased protein abundance for fermentation pathways in the four-species biofilm, whereas *Paenibacillus* increased protein abundance in similar pathways. The opposite regulation suggests competitive exclusion in anaerobic zones due to limited resources. This type of exclusion, creating niche separation between community members, is one of the expected structural arrangements emerging from competitive interactions^39,40^. The niche exclusion is supported by Liu *et al*.^15^ who observed that *Paenibacillus* would comprise the bottom and middle layer of the four-species biofilm, limiting *Xanthomonas*, *Stenotrophomonas* and *Microbacterium* to the top layer.

The observed pathway shifts in nitrogen metabolism from *Xanthomonas* and *Paenibacillus* support the niche separation. *Xanthomonas* had a significantly decreased abundance of the respiratory nitrate reductase in the four-species biofilm, while an increased abundance was observed for *Paenibacillus*. Measurements of nitrate in the outlet media showed that approx. 80% of the nitrate in the medium was removed by the biofilm, suggesting that nitrate is becoming a limited resource. As nitrate becomes limited, *Xanthomonas* is out-competed from the anaerobic regions, enforcing the niche separation. Observations from the nitrogen metabolism of *Paenibacillus* suggested that *Paenibacillus* further accumulated the nitrogen by assimilation through the NADP-specific glutamate dehydrogenase (Gln, Glu, Asp and Asn Biosynthesis). As *Paenibacillus* acquires nitrogen through ammonia assimilation, it would benefit from active degradation of amino acids by e.g. *Xanthomonas*, as this would provide additional ammonia for assimilation.

The opposing trends in branched-chain amino acid metabolism indicated that *Xanthomonas* and *Paenibacillus* were potentially facilitating each other by cross-feeding. Hansen *et al*.^14^ has previously shown by transcriptomics on batch-cultivated biofilms that the expression profile changed for both branched-chain and aromatic amino acid metabolism when *Xanthomonas* was cultured in the four-species biofilm, compared to dual and single species biofilm. Uniquely, co-cultivation of *Paenibacillus* and *Xanthomonas* caused the largest shift in the expression profile of *Xanthomonas*, verifying that these two species are interacting in the biofilm. In accordance with these results we found that *Paenibacillus* and *Xanthomonas* have opposite pathway regulation patterns and that *Xanthomonas* decreased branched-chain amino acids metabolism, when participating in the four-species biofilm. Whereas Hansen *et al*.^14^ did not elaborate on the cause for the altered metabolism, our data suggests that *Xanthomonas* is able to cross-feed on the branched-chain amino acids produced by *Paenibacillus*. Down regulation in energy-demanding amino acid biosynthesis pathways in *Xanthomonas* could partially explain the higher yield of biofilm formation for the community compared to the single species populations. Division of labour with respect to the production of amino acids has in other studies resulted in increased fitness of the community^41,42^.

The applied pathway analysis was unable to clearly indicate how participation in the four-species biofilm affected metabolic activity of *Stenotrophomonas* or how it interacted with the other species. Noticeably, a significant reduction in ribosome related proteins was observed in the pathway analysis of *Stenotrophomonas* when participating in the four-species biofilm, indicating hampered growth. In support, Liu *et al*.^15^ showed that *Stenotrophomonas* was reduced in biomass when participating in the four-species biofilm. The reason for its reduced growth in the four-species biofilm could not be deduced from the proteomic profiles, as no metabolic trends could be deduced from SCA proteins. This lacking trend could suggest that *Stenotrophomonas* does not adjust according to other species, but is simply reduced in growth through competition.

Combined, these observations contribute to the elucidation of the highly complex processes and interactions shaping the development of a biofilm community. The pathway shifts in *Paenibacillus*, going from central carbohydrate metabolism to fermentation and nitrogen metabolism, show that *Paenibacillus* completely changes its metabolic lifestyle when it is located in the four-species biofilm. Similarly, the metabolism of *Xanthomonas* appears to adjust due to the changed phenotype of *Paenibacillus*. These observations emphasize the need for continued co-culture studies to understand microbial life, as the life-style of the four-species biofilm cannot be predicted from observations of single species.

Proteomics assessment of microbial communities is becoming an increasingly more utilized approach to describe the underlying mechanisms influencing community development and function. However, some limitations still restrict the effectiveness of proteomics as a method. By example, as *Microbacterium* only comprised 1% of the cells in the four-species biofilm, it was not possible to reach sufficient proteome coverage to study it in the four-species biofilm. The dynamic range from the least to the most abundant protein in the sample limits the number of identified proteins from the low abundant species making it exceedingly difficult to obtain better coverage^43^.

An additional limitation is the genomic overlap between different phyla, genera and species, which hampers quantification and the taxonomical resolution of identified proteins^44,45^. Proteins with shared peptides can be indistinguishable and are therefore clustered across taxonomies as protein-group entries. Different approaches to obtain better taxonomic resolution have been presented in earlier studies, including e.g. DBToolKit^46^ for single proteome trimming, the ‘split by taxonomy’ function^47^ and ‘reduce to lowest common ancestor’^48^. Martens *et al*.^46^ developed the DBToolKit to prepare peptide databases from single proteomes for MS database searches, with the possibility of modifying resulting peptide databases with e.g. N-terminal ragging and removal of redundant peptides. Grassl *et al*.^47^ implemented the ‘split by taxonomy’ function in MaxQuant to obtain genus and partial species level resolution when describing the microbial diversity of the oral microbiome. Belstrøm *et al*.^48^ reduced available genomes to the lowest common ancestor to distinguish bacterial genera and species from peptides. Brooks *et al*.^49^ obtained strain-resolved proteomic profiles of two *Citrobacter* strains in fecal samples by utilizing strain-resolved community meta-genomic profiles of the two strains^49^. We developed a new pipeline to remove shared peptides in proteins from different species in smaller model communities. The pipeline is simple to use and applicable to the most commonly used search engines for MS data analysis. Compared to previous tools, e.g. DBToolKit, it provides visualisation of species overlap between reference proteomes, descriptive data of the proteome overlap and trimmed proteins and it prepares trimmed reference proteomes. The resource is accessible as an R script (Supplementary R-script 1). The approach enhanced taxonomic resolution of identified proteins with only minimal loss in the number of identified proteins and it provides more accurate quantitative information for identified proteins. The correlations between replicates were slightly impaired by removal of the overlapping peptides from the reference proteomes, but this could possibly be circumvented by further chromatographic peptide fractionation to identify more proteins, as well as enhancing the performance of the mass spectral normalization algorithms. A more in-depth proteome could additionally provide better pathway coverage, leading to higher confidence in pathway identification. As meta-proteomics is being applied more frequently to complex environmental samples, such as sea water^45^, waste-water treatment plants^9^, composting plants^50^, and soil^51^, future approaches to handle meta-proteomics data should evaluate the effect of gene-overlap on the analysis, as well as take steps to handle potential biases.

## Materials and Methods

### Cell cultures

Bacterial isolates were streaked on tryptic soy agar (TSA) plates (15 g/L agar and 30 g/L tryptic soy broth, VWR). Plates were incubated for 48 hrs at 24 °C, until single colonies could be picked. Single colonies were inoculated in 5 mL tryptic soy broth (TSB) (30 g/L tryptic soy broth, VWR) in test tubes and incubated overnight at 24 °C with shaking at 250 rpm. Subsequently, these cultures were serially diluted in 30 mL TSB to obtain exponential phase cultures. The 30 mL cultures were incubated at 24 °C with shaking at 250 rpm overnight. The following day the exponential phase cultures were selected (OD 0.15–0.6) and diluted in TSB to OD 0.15 before use.

### Preparing the Drip Flow Reactor

The drip flow reactor (DFR) (Biosurface Technologies, USA) was equipped with a standard microscope glass slides in each chamber. A 10 L blue cap bottle containing seven liter TSB was sealed with a GL45 SecurityLid (VWR), equipped with a stainless steel pipe and a 0.2 µm PTFE air-filter (VWR). The DFR, media, inlet and outlet tubing were autoclaved before use. The media bottle was connected to a Watson-Marlow 205 S pump equipped with Marprene tubes (1 mm inner diameter) (Watson-Marlow), which supplied media to the DFR. Media bottle and pump were connected through silicone tubes (1 mm inner diameter, Mikrolab, Denmark). The pump and the DFR were connected through silicone tubes (0.5 mm inner diameter, Mikrolab, Denmark) fitted with 21 G × 1″ 0.8 mm × 25 mm syringes (BD MicrolanceTM). Silicone tubing between media bottle, pump and syringe was connected using male and female luer lock fittings.

### Running the Drip Flow Reactor

Each chamber outlet channel was blocked by clamps and each reactor chamber was inoculated with 20 mL OD 0.15 adjusted culture. For the four-species community 5 mL of each species was mixed together and added to the chamber. The DFR was horizontally incubated at room temperature for four hours to allow cell attachment to the glass slides. After the cell attachment phase, the DFR was placed on a tilted stand (with a 10° downward tilt). Non-attached cells and media were removed from the DFR. Fresh media was added at a constant flow of 0.4 mL/min. The reactor was left at room temperature until biofilm harvest. Glass slides were removed from the reactor and biofilm was scraped off into a protein extraction buffer (200 mM Tris HCl pH 7.6, 200 mM NaCl, 1 mM EDTA, 5% glycerol and 20 mM DTT), instantly frozen in liquid nitrogen and stored at −18 °C until protein extraction.

### Biological replicates included in the study

Three biological replicates were included for *Microbacterium* and five biological replicates were included for *Xanthomonas*, *Stenotrophomonas*, *Paenibacillus* and the four-species biofilm. Each individual biological replicate was produced from an individual set of starter cultures, and each starter culture was made from a single CFU. As the quantity of biofilm varied between single species, multiple technical replicate slides were pooled for single species *Paenibacillus* and *Microbacterium* for each biological replicate. For both *Paenibacillus* and *Microbacterium*, three technical replicate biofilms were pooled to ensure sufficient biomass for protein extraction. Only one technical replicate was used per biological replicate of *Xanthomonas*, *Stenotrophomonas* and the four-species biofilm.

### Protein extraction and digestion

Biofilm cells were lysed with a Q500 sonicator (Qsonica, Newtown, USA) for 4 min at 30% amplitude in the protein extraction buffer. The sonicated samples were centrifuged at 10000 *g* and soluble proteins in the supernatants were precipitated by adding ice-cold acetone to a final concentration of 80%. Samples were precipitated over night at −18 °C. Precipitated proteins were re-suspended in a urea solution (8 M urea, 50 mM ammonium bicarbonate-buffer, pH 8), protein concentration was estimated via Bradford assay, and samples were diluted to 5 μg/μL, reduced with 5 mM DTT for one hour, and alkylated with 20 mM iodoacetamide for one hour in the dark. Prior to trypsin digestion protein concentration was measured in all samples and 30 μg protein material was used for digestion. The samples were four fold diluted in 50 mM ammonium bicarbonate pH 8, and then incubated overnight by trypsin (1:100 trypsin-to-protein ratio) at room temperature with horizontal shaking at 500 rpm. Inactivation of trypsin was achieved by adding trifluoroacetic acid (TFA) to 2% and debris was removed by centrifugation (10000 g, 10 min). The tryptic peptides were fractionated on SDB-RPS STAGE- tips^52^. Acidified peptides were loaded on the filter discs by centrifugation at 1000 g for 5 min, and the filter unit was washed twice using 0.1% TFA. Peptides were eluted into three fractions using elution buffer 1: 100 mM ammonium formate, 40% acetonitrile, 0.5% formic acid; elution buffer 2: 150 mM ammonium formate, 60% acetonitrile, 0.5% formic acid; elution buffer 3: 5% ammonium hydroxide, 80% acetonitrile. Volatile salts were evaporated from the eluted fractions in a speedvac and the desalted pellets were re-dissolved in 0.1% formic acid. Assuming that peptides elute evenly in each fraction^52^, equal amounts of each fraction were subjected to mass spectrometry analysis, aiming for a theoretical loading of 1.5 μg peptides per fraction.

### Mass spectrometry

The samples were analyzed by liquid chromatography tandem mass spectrometry (LC-MS/MS) and data were recorded in a data dependent manner, automatically switching between MS and MS/MS acquisition, on a Q-Exactive (Thermo scientific, Bremen, Germany). An EASY nLC-1000 liquid chromatography system (Thermo scientific, Odense, Denmark) was coupled to the mass spectrometer through an EASY spray source and peptide separation was performed on 50 cm EASY-spray columns (Thermo scientific) with 2 µm size C18 particles and inner diameter of 75 µm. Mobile phase consisted of solvents A (0.1% formic acid) and B (80% acetonitrile in 0.1% formic acid). The initial concentration of solvent B was 6%, and hereafter gradients were applied to reach the following concentrations: 14% B in 37 min, 25% B in 42 min, 38% B in 21 min, 60%B in 20 min, 95% B in 3 min and 95% B for 7 min. The total length of the gradient was 130 min. The full scans were acquired in the Orbitrap with a resolution of 70000 and a maximum injection time of 20 ms was applied. For the full scans, the range was adjusted to 350–1500 m/z. The top ten most abundant ions from the full scan were sequentially selected for fragmentation with an isolation window of 1.6 m/z^53^, and excluded from re-selection for a 30 sec. time period. For the MS/MS scans the resolution was adjusted to 17500 and maximum injection time of 60 ms. The ions were fragmented in a higher-energy collision dissociation (HCD) cell with normalized collision energy (NCE) of 28%.

### Generating non-overlapping reference proteomes for multi-species analysis

Reference genomes have been prepared as part of an earlier investigation and are available online; PRJEB18431 (*Xanthomonas retroflexus*), PRJEB15265 (*Microbacterium oxydans*), PRJEB15263 (*Stenotrophomonas rhizophila*), PRJEB15262 (*Paenibacillus amylolyticus*). Reference proteomes were based on the genomes which were annotated with the RAST database^29,30^. Each proteome was loaded into the statistical software environment R with SeqinR v3.3-1^54^, and *in silico* trypsin digested using the Cleaver v1.10.2 package. No missed cleavages were allowed. Resulting peptides were filtered for a minimum length of 7 amino acids, and were compared between all four reference proteomes for identical string matches. Any peptide found in two or more species was removed from all reference proteomes, by deleting the amino acid strings and concatenating the adjacent fragments. This resulted in trimmed reference proteomes with protein sequences containing only the trypsin-digested peptides unique to each species.

### Analysis of mass spectrometry data

Data analysis was carried out by analysing each species separately; single-species files were analysed together with the four-species biofilm files and the genome of the specific species. The acquired raw data was analysed using MaxQuant version 1.5.5.1^55^ with the inbuilt Andromeda search engine^56^. Mass tolerance was set to 4.5 ppm (parent ions) and 20 ppm (fragment ions); a maximum of 2 missed tryptic cleavages were permitted. Methionine oxidation and protein N-terminal acetylation were selected as variable modifications, and carbamidomethylation of cysteines as a fixed modification. A minimum length of 7 amino acids per peptide was required. A target decoy search approach with the default MaxQuant setting of 1% FDR was applied for identification at both peptide and protein levels^55^. Normalization was performed with the label free quantification (MaxLFQ) algorithm^57^ in MaxQuant using a required LFQ minimum ratio count of two. Quantification also required a minimum ratio count of two, allowing quantification only on unique and razor peptides. The ‘match between runs’ function was applied to enhance protein identification. The mass spectrometry data from each of the three fractions from each biological replicate were combined to a single biological replicate in MaxQuant before quantification.

The mass spectrometry proteomics raw data and results from analysis by MaxQuant have been deposited to the ProteomeXchange Consortium via the PRIDE^58^ partner repository with the dataset identifier PXD006281.

### Protein analysis and statistics

Statistical analysis and data visualisation were performed with the Perseus software v1.5.3.2^59^ and in R v3.3.0 using ggplot2 v2.1.0^60^. Regulated proteins were identified using a modified Welsh t-test with an S0 parameter of 1^61^, a permutation based FDR cut-off of 0.05 and valid values in at least 60% of the samples in both single and multi-species conditions (Supplementary Table S3). Supplementary Fig. 7 shows protein overlap of quantifiable proteins between the single and four-species biofilms. Regulated pathways were identified using Fishers exact test and Benjamini Hochberg FDR correction^62^ was used for correcting for multiple hypothesis testing.

### Data Availability

All mass spectrometry data generated and analysed during the current study are available in online repositories as stated throughout materials and methods. Biological data of e.g. nitrate measurements and CFU counts is available from the corresponding author on reasonable request.

### Performing counts of colony forming units and crystal violet staining on biofilms

Counts on colony forming units (CFU) in the four-species biofilm were performed by scraping off the biofilm from glass slides into 1 mL phosphate buffered saline solution (1xPBS). Biofilm was disrupted by vortexing the solution five times for 30 sec with 30 sec rest between each round of disruption. Disrupted biofilm samples were serial diluted and plate spread on TSA plates complemented with 40 μg/mL congo red (Fluka) and 20 μg/mL coomassie brilliant blue G250 (Sigma-Aldrich). Plates were incubated for two days at room temperature and one day at 4 °C. After incubation, *Paenibacillus* and *Microbacterium* could be counted selectively due to their distinct morphology appearance, and only a combined *Xanthomonas* and *Stenotrophomonas* count could be obtained as these two could not be distinguished based on morphology. Counts of *Xanthomonas* and *Stenotrophomonas* were therefore achieved by selectively counting *Xanthomonas* on plates complemented with kanamycin (50 mg/ml, final concentration), as *Xanthomonas* was the only species resistant to kanamycin.

Biofilm biomass from mono- and the four-species biofilms was quantified using a modified crystal violet (CV) assay. Glass slides with attached biofilm were removed from the DFR and biofilms were fixated on the slides by submerging the slides in 99% methanol for 15 min at room temperature. After incubation, slides were air-dried, and then stained by submerging the slides in a 1% (w/v) CV solution. Slides were stained for 20 min at room temperature. After staining, slides were rinsed three times in 1xPBS until unbound CV had been removed. Biofilms were de-stained by submerging each slide in a 50 mL centrifugation tube containing 40 mL of 96% ethanol for 30 min. Absorbance measurements on the de-staining solution were used to quantify biofilm biomass. Absorbance measurements was performed at 590 nm on a ELX 808IU Absorbance Microplate Reader (BioTek Instruments).

### Nitrate and ammonia measurements

Nitrate and ammonia measurements were performed on effluent media collected from the DFR right before biofilm harvest. Effluent media was collected for 5 min before harvest, and the effluent was filtered on 0.2 µm syringe filters (LLG Labware) to remove cells. Subsequently the ammonia concentration in the effluent was measured with the Ammonia Assay Kit (Sigma-Aldrich) according to manufacturers instructions and nitrate was measured with the Nitrite/Nitrate Assay Kit (Sigma-Aldrich).

## Electronic supplementary material

Supplementary Information
