# Supplementary material for: A meta-proteomics approach to study the interspecies interactions affecting microbial biofilm development in a model community
Source: Sci Rep. 2017 Nov 28;7:16483. doi: 10.1038/s41598-017-16633-6 (PMC5705676; doi:10.1038/s41598-017-16633-6)
Supplement: Supplementary file 1 — Supplementary Information [file 41598_2017_16633_MOESM1_ESM.pdf]

Supplementary Information

A meta-proteomics approach to study the interspecies interactions affecting microbial biofilm development in a model community

Jakob Herschend[1, #], Zacharias B. V. Damholt[2 #], Andrea M. Marquard[3], Birte Svensson[2], Søren J. Sørensen[1], Per Hägglund[2] and Mette Burmølle[1, +]
[1]Section of Microbiology, Department of Biology, University of Copenhagen, Denmark
[2]Department of Biotechnology and Biomedicine, Technical University of Denmark, Denmark
[3] Section for Immunology and Vaccinology, National Veterinary Institute, Technical University of Denmark, Denmark
[#]Authors contributed equally
[+]Corresponding author

Number of figures: 20 supporting figures
Number of tables: 4 supporting tables
Number of R-scripts: 1 supporting R-script

Correspondence: Mette Burmølle, Universitetsparken 15 Bldg. 1, 2100, Denmark.
+4540220069, burmolle@bio.ku.dk

Supplementary figures

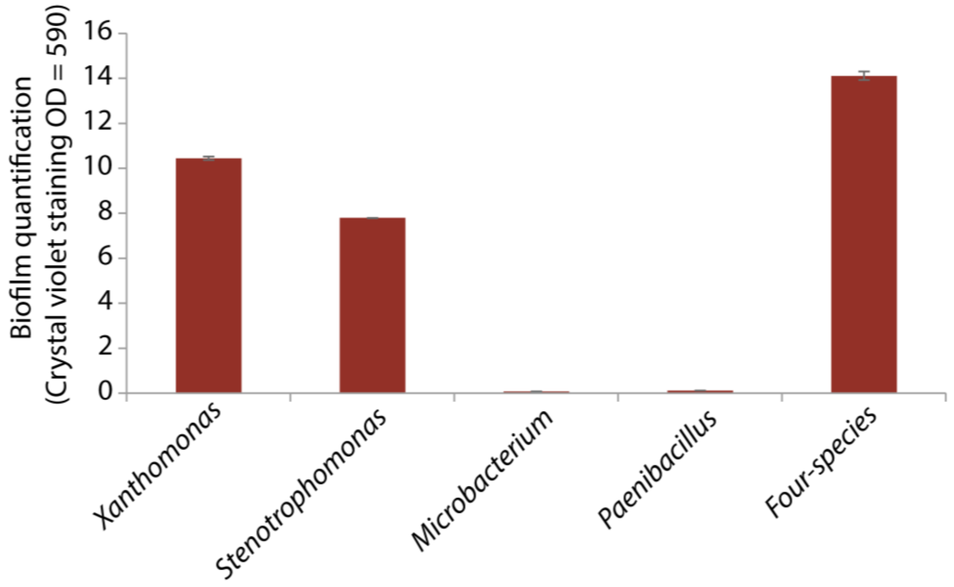

Supplementary fig. S1: Crystal violet staining of single- and four-species biofilm after 48 hrs growth. Data originates from a single biological replicate, with error bars indicating standard deviation of measured crystal violet (n=3).

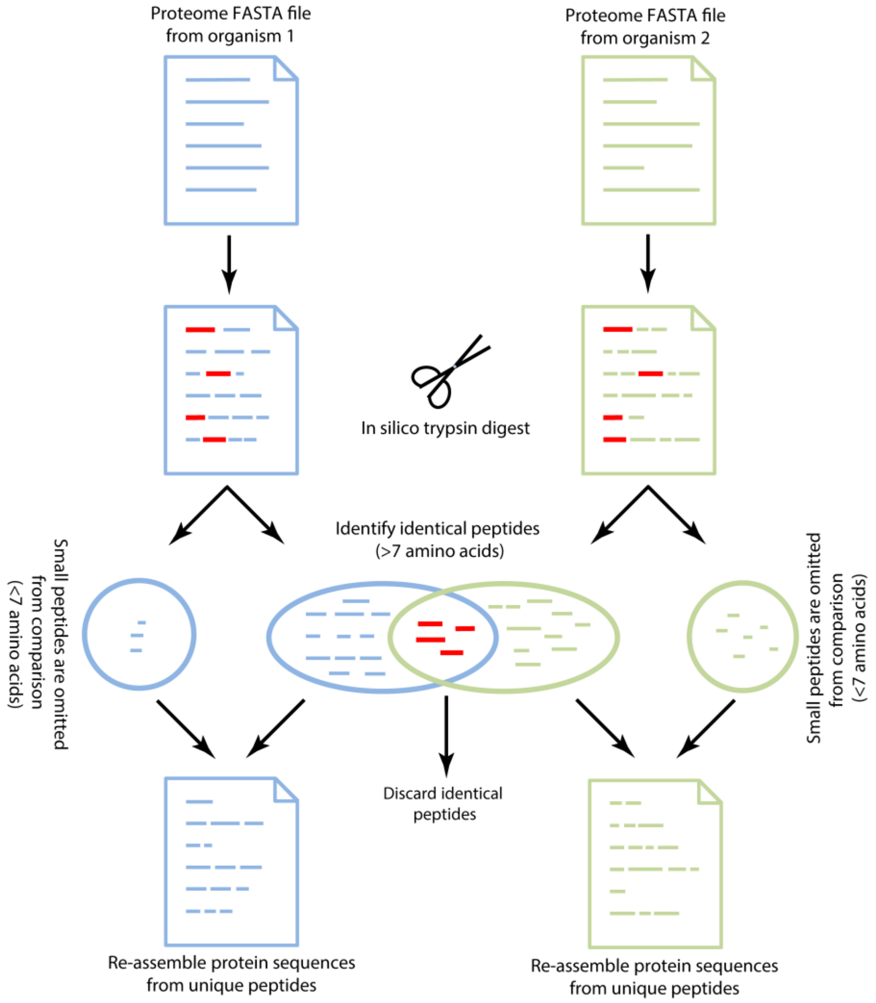

Supplementary fig. S2: Trimming procedure of reference proteomes. The reference proteomes are *in silico* digested with trypsin. Resulting peptides are binned according to their length. Peptides <7 amino acids are omitted from the analysis of sequence homology. Peptides >7 amino acids are compared to peptide sequences from all the other reference proteomes. Identical peptides from each reference proteome (indicated in red) are discarded. The remaining peptides >7 and peptides <7 are then re-assembled into protein sequences, which now only contain peptides that are unique to that organism.

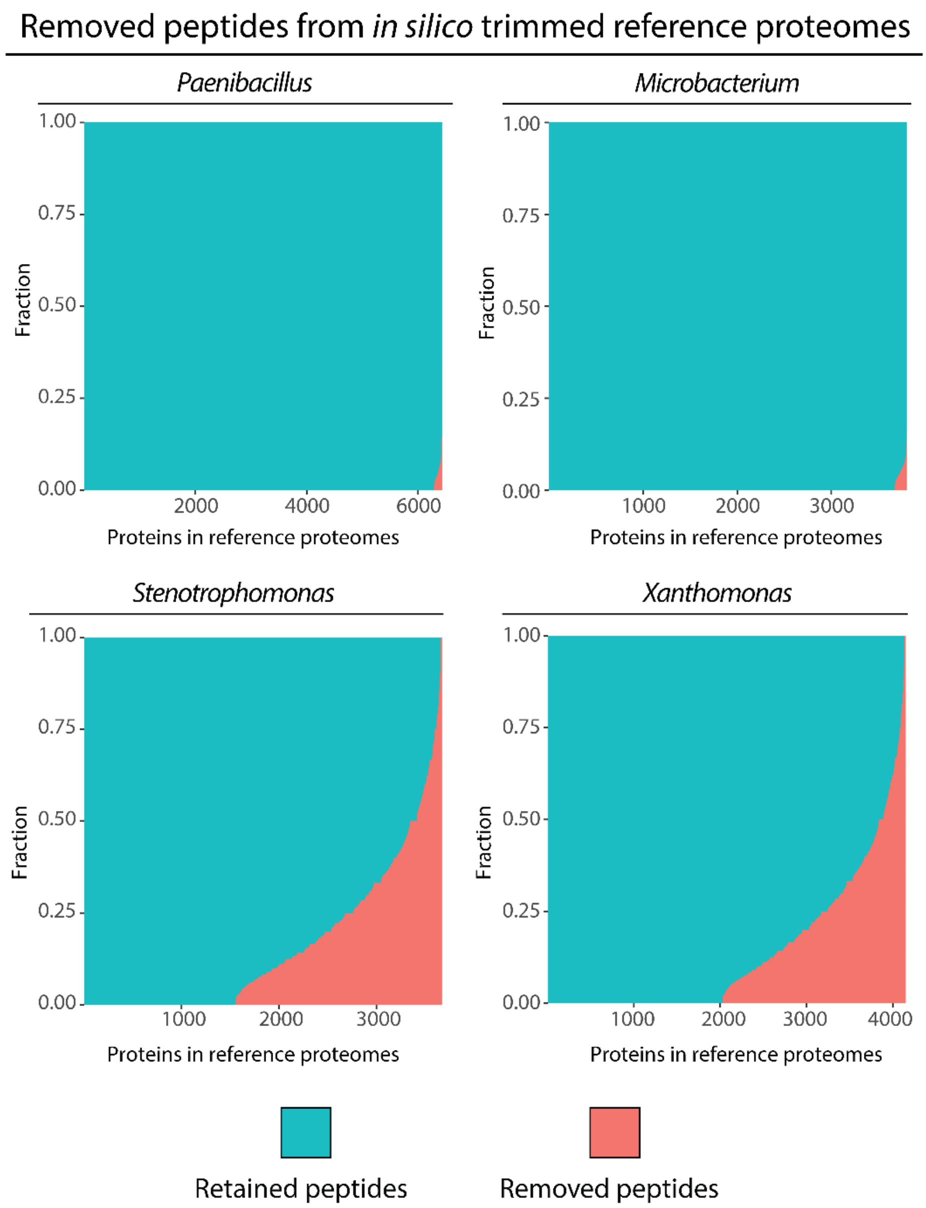

Supplementary fig. S3: Peptides retained or removed from reference proteomes. Reference proteomes were *in silico* digested with trypsin and the resulting peptides were filtered for a minimum length of 7 amino acids. These peptides were then compared between the four reference proteomes and any peptides shared between two or more species were removed from the reference proteomes. The retained peptides were then assembled back into proteins and the resulting reference proteomes now only contain peptides unique to each species.

Data analysis performed for preparation of *"Supplementary fig. S4: Effect of trimmed reference proteomes on protein identification in four-species biofilm"* and *"Supplementary fig. S5: Protein quantification with trimmed reference proteomes"*

The five biological replicates were analysed in MaxQuant with reference proteomes for all four species simultaneously included in the MaxQuant data analysis, using either trimmed or standard genome. Mass spectrometry data from four-species samples were analyzed using MaxQuant version 1.5.5.1 with the inbuilt Andromeda search engine, with mass tolerance set to 4.5 ppm (parent ions) and 20 ppm (fragment ions). A maximum of 2 missed tryptic cleavages were permitted. Methionine oxidation and protein N-terminal acetylation were selected as variable modifications, and carbamidomethylation of cysteines as a fixed modification. A minimum length of 7 amino acids per peptide was required. A target decoy search approach with the default MaxQuant setting of 1% FDR was applied for identification at both peptide and protein levels. Normalization was performed with the label free quantification (MaxLFQ) algorithm in MaxQuant using a required LFQ minimum ratio count of two. Quantification also required a minimum ratio count of two, allowing quantification only on unique and razor peptides. The match-between-runs function was not applied when analyzing data with all four reference proteomes simultaneously. The fraction normalization was not applied but the mass spectrometry data from each of the three fractions from each biological replicate were combined to a single biological replicate in MaxQuant before quantification.

Application of trimmed reference proteomes lead to a significant reduction in the mean number of identified proteins which can be resolved at species level (Unique) and those that cannot be resolved at species level (Shared). For the individual species the average number of proteins which could be resolved at species level was significantly reduced for *Xanthomonas* with application of the trimmed reference proteomes, but not for *Paenibacillus* and *Stenotrophomonas*. Proteins which could not be resolved at species level with the trimmed reference proteomes were identified as proteins with peptide miss-cleavages. Combined from the five biological replicates, application of the trimmed reference proteomes lead to a reduction of identified proteins with species level resolution of 18 proteins for *Paenibacillus* and 4 for *Xanthomonas.* However, for *Stenotrophomonas* the total number of identified proteins increased with 110 proteins, see supplementary fig. 4a-c.

Application of trimmed reference proteomes caused a significant change in average protein intensity for proteins trimmed by the pipeline for both Xanthomonas and Stenotrophomonas, but not for Paenibacillus. The change in average protein intensity was significant for both raw protein intensities and after normalisation by the MaxQuant LFQ normalisation algorithm, see supplementary fig. 5a-c.

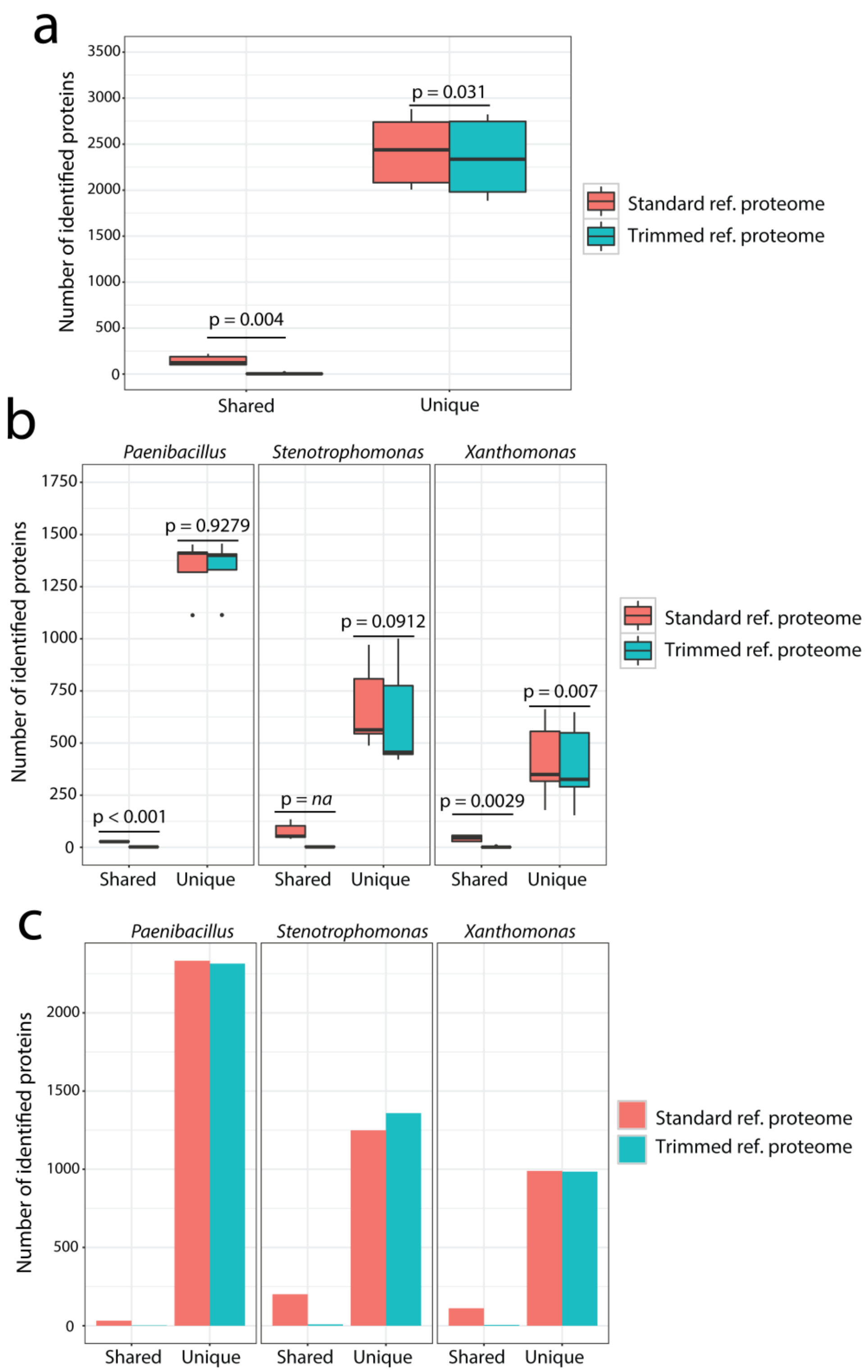

Supplementary figure S4a-c: Effect of trimmed genomes on protein identification in four-species biofilm. Protein identification with different genome types. Shared proteins refer to identified proteins that cannot be resolved at species level. Unique refers to identified proteins which could be resolved at species level. Proteins which could not be resolved at species level with the trimmed reference proteomes solely consists of peptides with missed cleavages. Statistical difference was inferred using paired t-test. P-values marked

with *na* indicates that t-test was not performed due to lacking proteins in some groupings. P-values below 0.05 are considered significant.
a) Number of identified proteins from the four-species biofilm across the five biological replicates using standard or trimmed reference proteomes for mass spectrometry data analysis in MaxQuant. Application of the trimmed reference proteomes significantly lowers the number of both shared and unique identified proteins across all five biological replicates.
b) Number of identified proteins for the three major species in the four-species biofilm across the five biological replicates when using standard or trimmed reference proteomes for data analysis. Application of trimmed reference proteomes lead to a significant reduction in the number of species unique proteins across all five biological replicates for *Xanthomonas*, but no significant change was observed for *Paenibacillus* and *Stenotrophomonas*.
c) Total number of identified proteins across the five biological replicates for each of the three major species in the four-species biofilm. With trimmed reference proteomes the number of species unique proteins was reduced with 18 and 5 for *Paenibacillus* and *Xanthomonas* respectively, but increased with 110 for *Stenotrophomonas*.

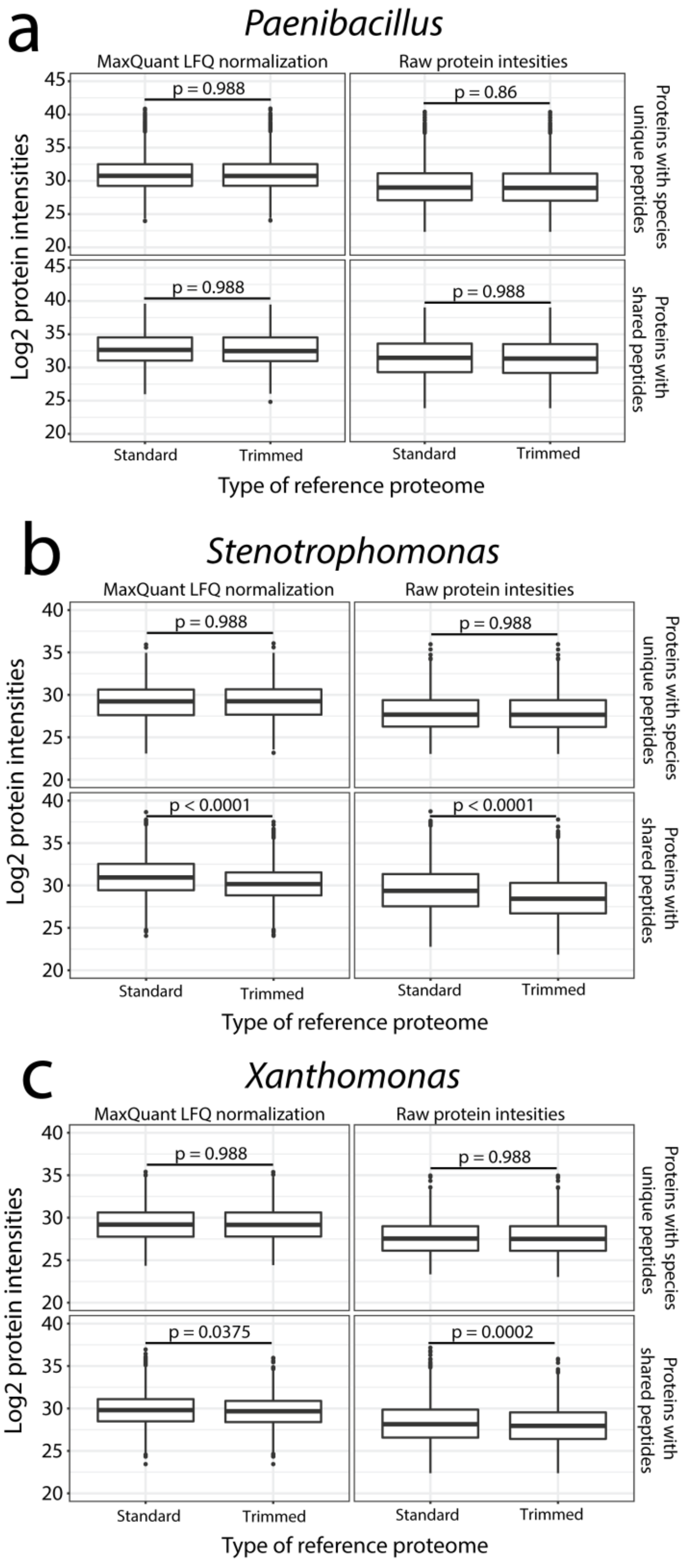
a
Paenibacillus
MaxQuant LFQ normalization
Raw protein intesities
p = 0.988
p = 0.86
p = 0.988
p = 0.988
Proteins with species unique peptides
Proteins with shared peptides
Log2 protein intensities
Standard
Trimmed
Type of reference proteome
b
Stenotrophomonas
MaxQuant LFQ normalization
Raw protein intesities
p = 0.988
p = 0.988
p < 0.0001
p < 0.0001
Proteins with species unique peptides
Proteins with shared peptides
Log2 protein intensities
Standard
Trimmed
Type of reference proteome
c
Xanthomonas
MaxQuant LFQ normalization
Raw protein intesities
p = 0.988
p = 0.988
p = 0.0375
p = 0.0002
Proteins with species unique peptides
Proteins with shared peptides
Log2 protein intensities
Standard
Trimmed
Type of reference proteome

Supplementary figure S5a-c: Protein quantification with standard and trimmed reference proteomes. Protein quantification of identified proteins for the three major species in four-species biofilm. Protein quantification grouped for both trimmed and standard reference proteomes, along with MaxQuant LFQ normalized protein intensities and raw protein intensities. 'Proteins with shared peptides' refers to the proteins which were *in silico* trimmed by the pipeline, and 'Proteins with species unique peptides' refers to proteins which were not trimmed by the pipeline. Statistical difference was inferred by a linear mixed effect model accounting for the random effect of biological replicates. P-values were FDR corrected. P-values are considered significant below 0.05.
a) Protein quantification of identified *Paenibacillus* proteins, with and without trimmed reference proteomes. Application of trimmed reference proteomes does not significantly affect average protein intensities of identified proteins.
b) Protein quantification of identified *Stenotrophomonas* proteins, with and without trimmed reference proteomes. Application of trimmed reference proteomes significantly altered mean protein intensities of proteins which were trimmed by the pipeline. The change in mean protein intensity was significantly changed for both raw intensities and MaxQuant LFQ normalised proteins.
c) Protein quantification of identified *Xanthomonas* proteins, with and without trimmed reference proteomes. Application of trimmed reference proteomes significantly altered the mean protein intensity of proteins which were trimmed by the pipeline. The change in mean protein intensity was significantly changed for both raw intensities and MaxQuant LFQ normalised proteins.

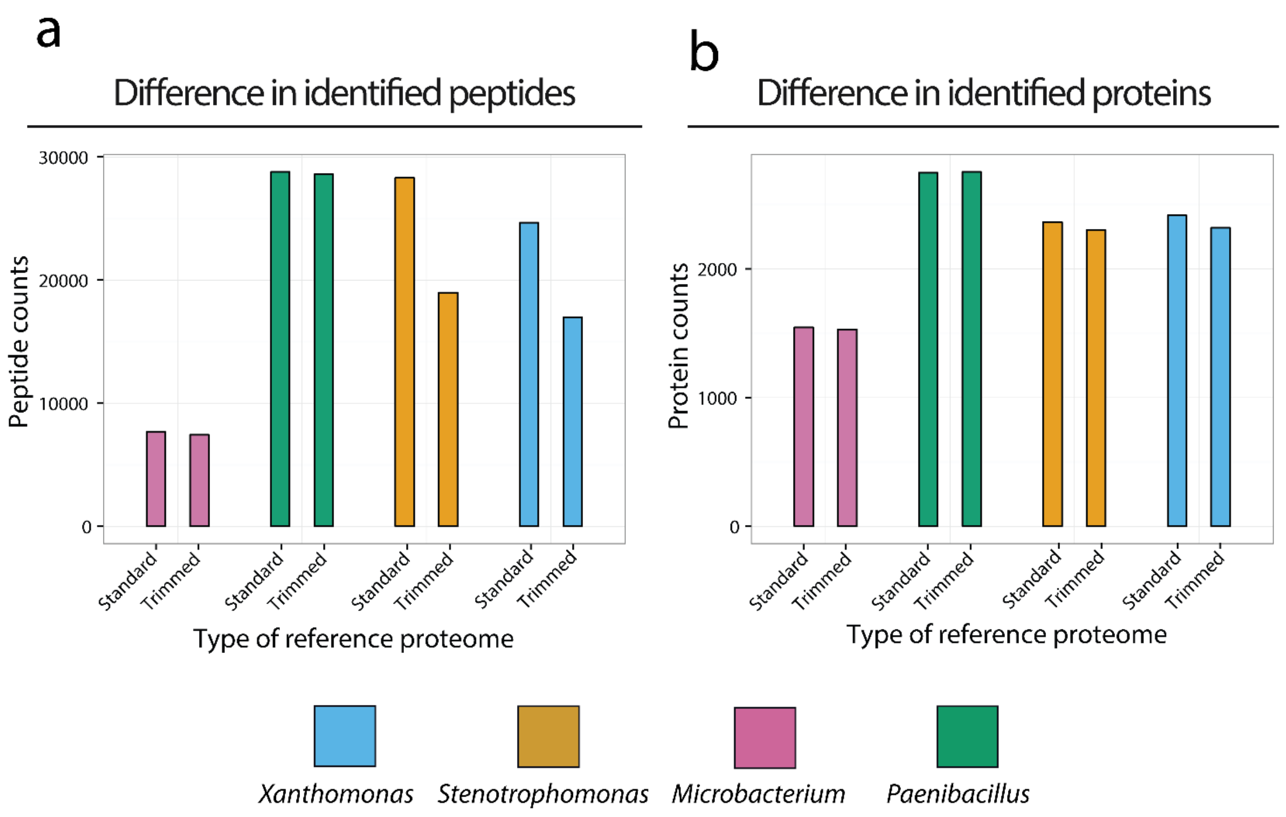

Supplementary fig. S6: Biofilm samples were analysed with either the standard reference proteomes (Standard) or the trimmed reference proteomes (Trimmed). Bars represents the combined protein counts for identified proteins in both single- and four species biofilms. a) Indicates the number of identified peptides with the standard or trimmed reference proteomes. Large reductions in the numbers of identified peptides were observed in *Stenotrophomonas* and *Xanthomonas*, when using the trimmed reference proteomes. b) Number of identified proteins from the MS data using the standard and trimmed reference proteomes. Though a large number of peptides were lost using the trimmed reference proteomes, there was only a small effect on the total numbers of identified proteins. Sixteen proteins were lost from *Microbacterium*, *Paenibacillus* gained 7 proteins, *Stenotrophomonas* lost 61 proteins and *Xanthomonas* lost 96 identified proteins when using the trimmed reference proteins.

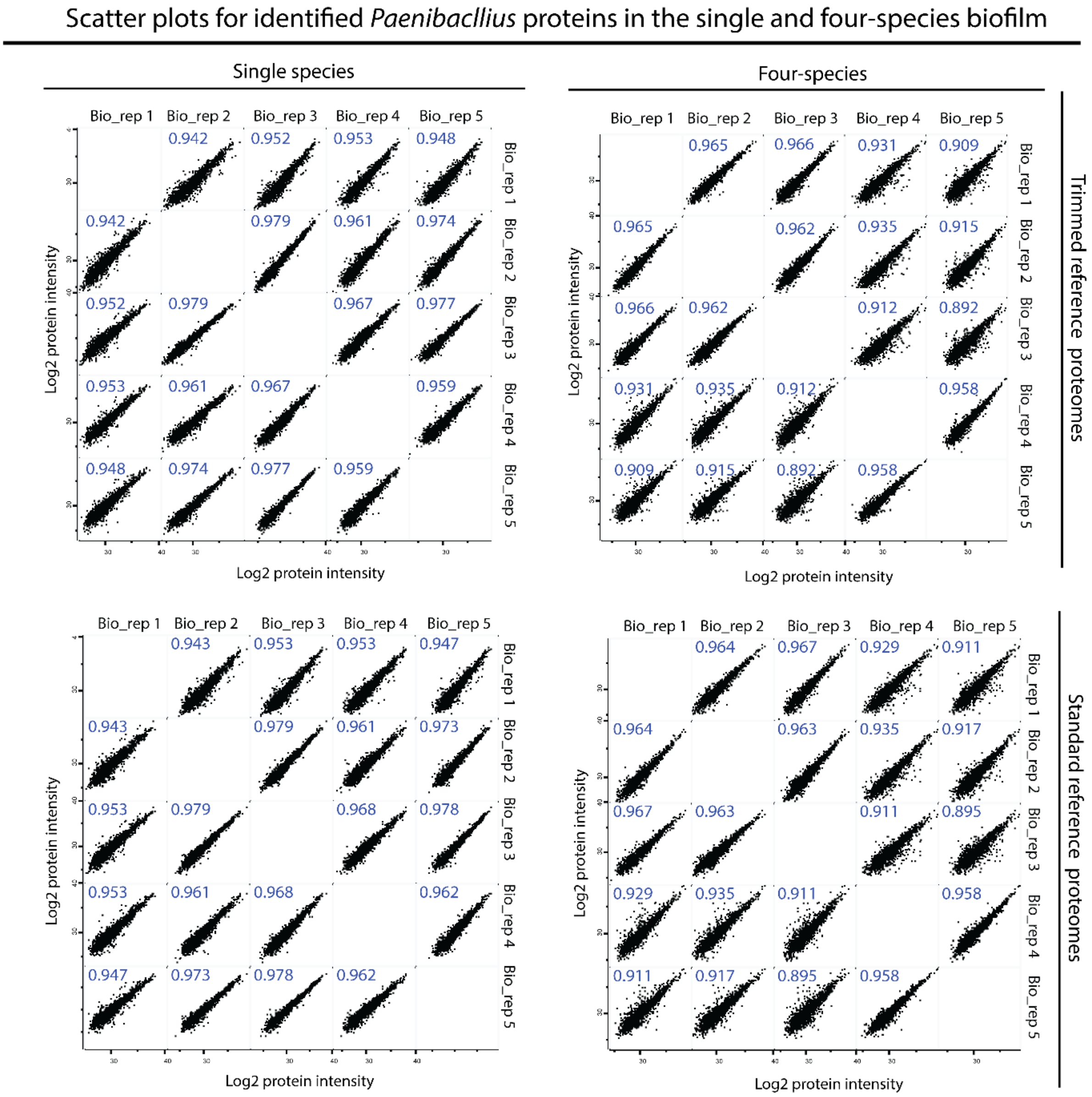

Supplementary fig. S7: Correlation of protein abundance profiles between five biological replicates of *Paenibacillus* in both single and four-species biofilm. Sample correlation was performed using Pearson correlation. Correlation plots are presented for data with both standard and trimmed reference proteomes.

Scatter plots for identified *Stenotrophomonas* proteins in the single and four-species biofilm

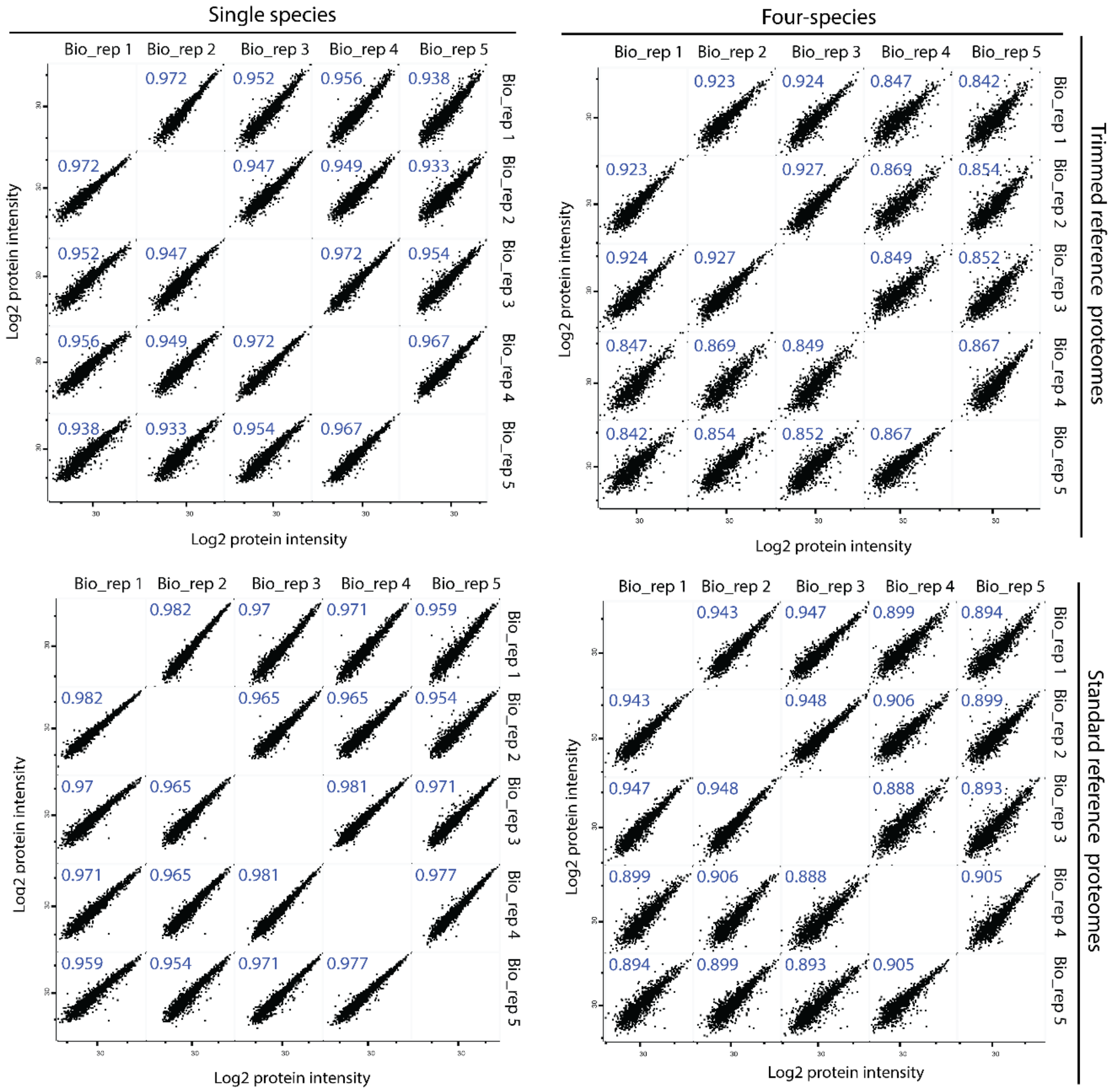

Supplementary fig. S8: Correlation of protein abundance profiles between five biological replicates of *Stenotrophomonas* in both single and four-species biofilm. Sample correlation was performed using Pearson correlation. Correlation plots are presented for data with both standard and trimmed reference proteomes.

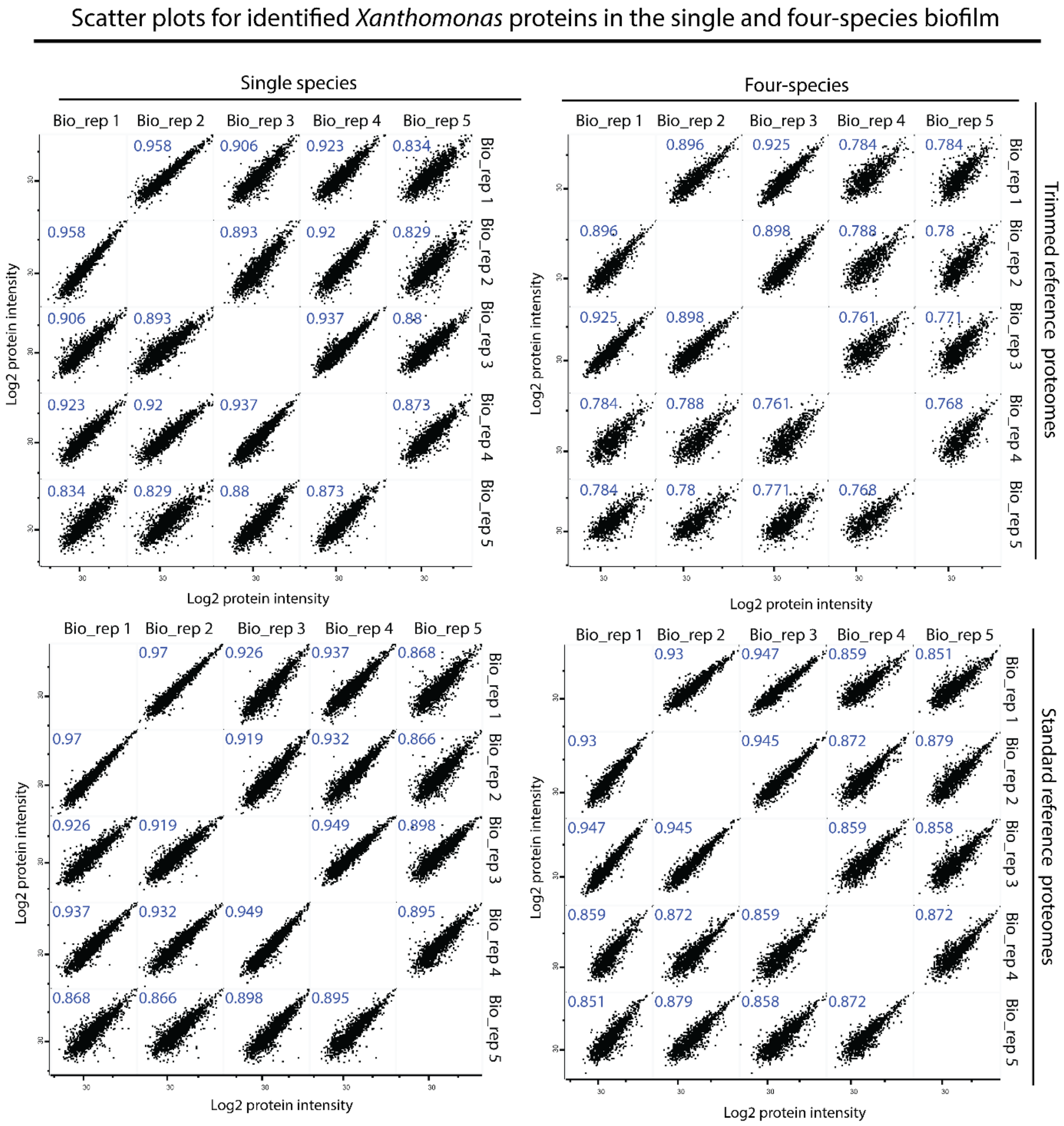

Supplementary fig. S9: Correlation of protein abundance profiles between five biological replicates of *Xanthomonas* in both single and four-species biofilm. Sample correlation was performed using Pearson correlation. Correlation plots are presented for data with both standard and trimmed reference proteomes.

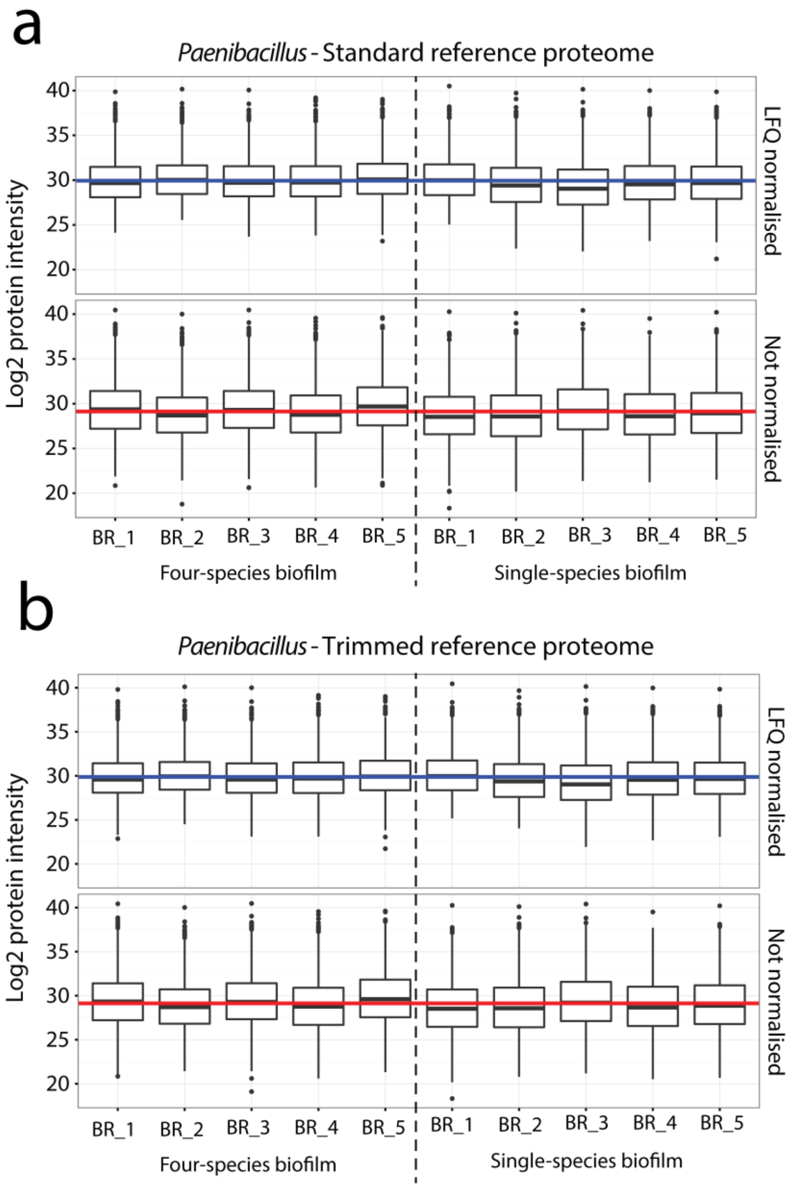

Supplementary fig. S10: Mean log2 protein intensities for identified *Paenibacillus* proteins in all biological replicates (BR_1-5) of single and four-species biofilm, with and without MaxQuant LFQ normalisation. Blue line indicates the log2 mean protein intensity for LFQ normalised proteins across both single and four-species samples. Red line indicates the log2 mean protein intensity for proteins without any normalization across both single and four-species samples. a) Mean Log2 protein intensities for *Paenibacillus* in the single and four-species biofilm using standard reference proteomes.

b) Mean Log2 protein intensities for proteins *Paenibacillus* in the single and four-species biofilm using trimmed reference proteomes.

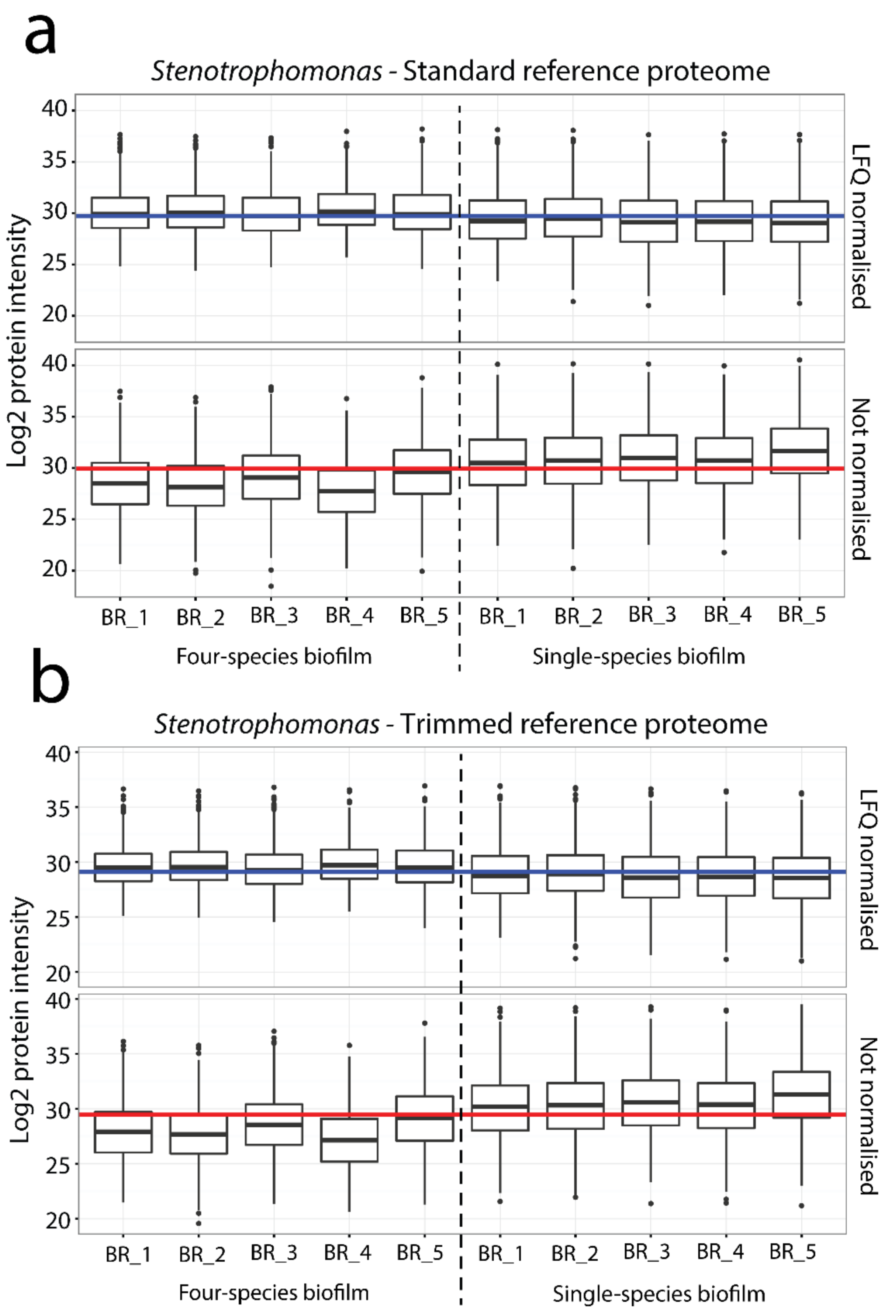

Supplementary fig. S11: Mean log2 protein intensities for identified *Stenotrophomonas* proteins in all biological replicates (BR_1-5) of single and four-species biofilm, with and without MaxQuant LFQ normalisation. Blue line indicates the log2 mean protein intensity for LFQ normalised proteins across both single and four-species samples. Red line indicates the log2 mean protein intensity for proteins without any normalization across both single and four-species samples. a) Mean Log2 protein intensities for *Stenotrophomonas* in the single and four-species biofilm using standard reference proteomes.
b) Mean Log2 protein intensities for proteins *Stenotrophomonas* in the single and four-species biofilm using trimmed reference proteomes.

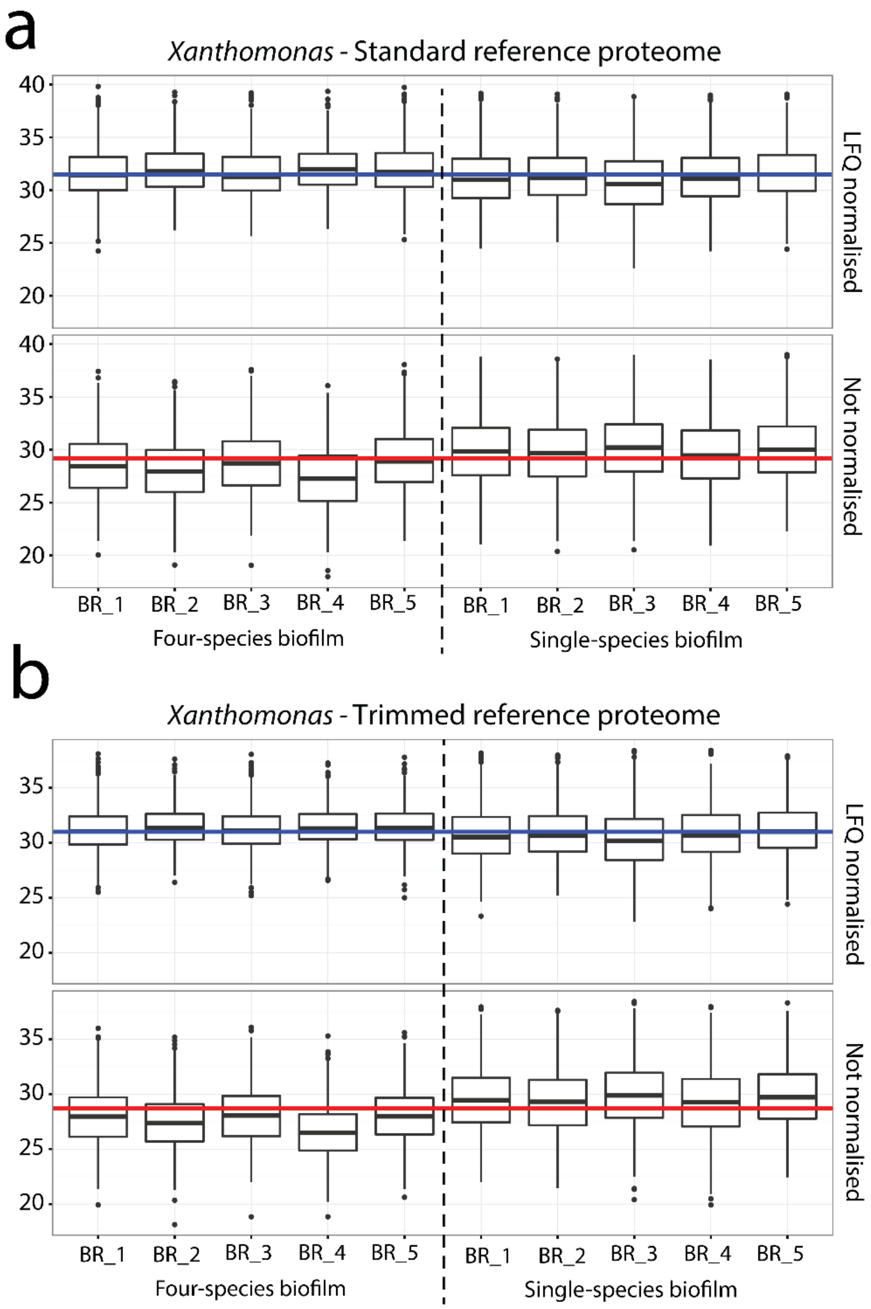

Supplementary fig. S12: Mean log2 protein intensities for identified *Xanthomonas* proteins in all biological replicates (BR_1-5) of single and four-species biofilm, with and without MaxQuant LFQ normalisation. Blue line indicates the log2 mean protein intensity for LFQ normalised proteins across both single and four-species samples. Red line indicates the log2 mean protein intensity for proteins without any normalization across both single and four-species samples. a) Mean Log2 protein intensities for *Xanthomonas* in the single and four-species biofilm using standard reference proteomes.
b) Mean Log2 protein intensities for proteins *Xanthomonas* in the single and four-species biofilm using trimmed reference proteomes.

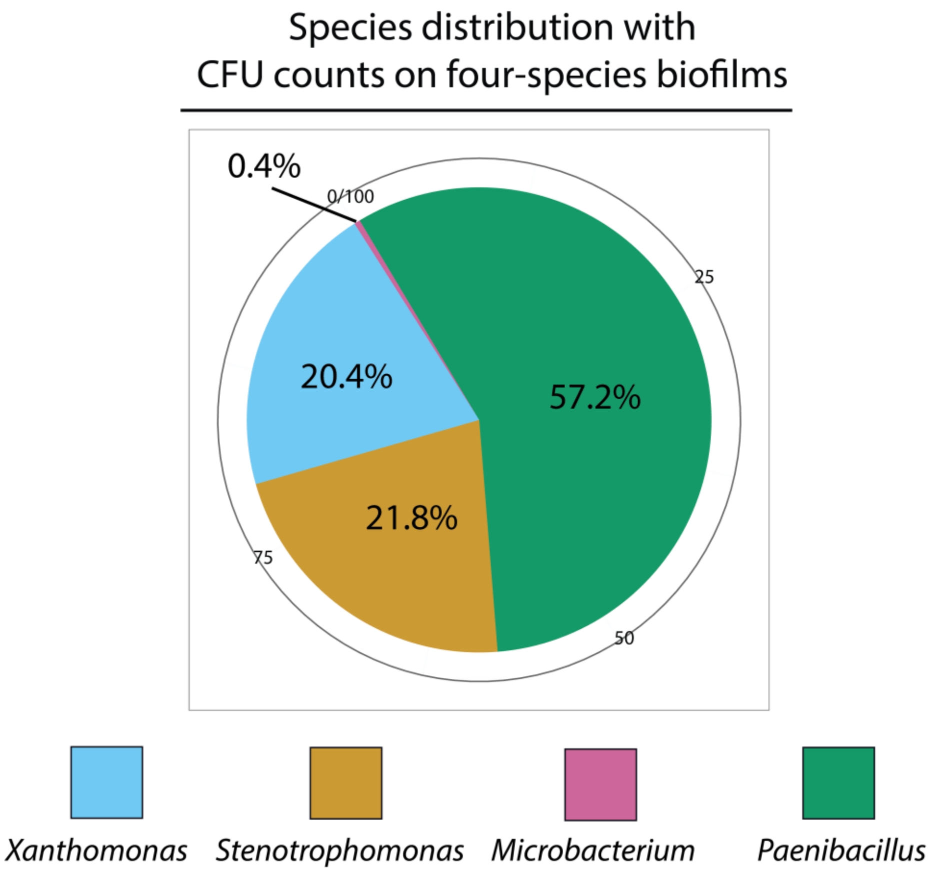

Supplementary fig. S13: Species distribution of the four species in the four-species biofilm based on CFU counts. The two days old biofilm was scraped of the glass slide and vortexed in 1xPBS. Biofilm sample was diluted and plated on TSA plates complemented with 40µg/ml Congo red and 20 µg/ml coomassie brilliant blue G250.

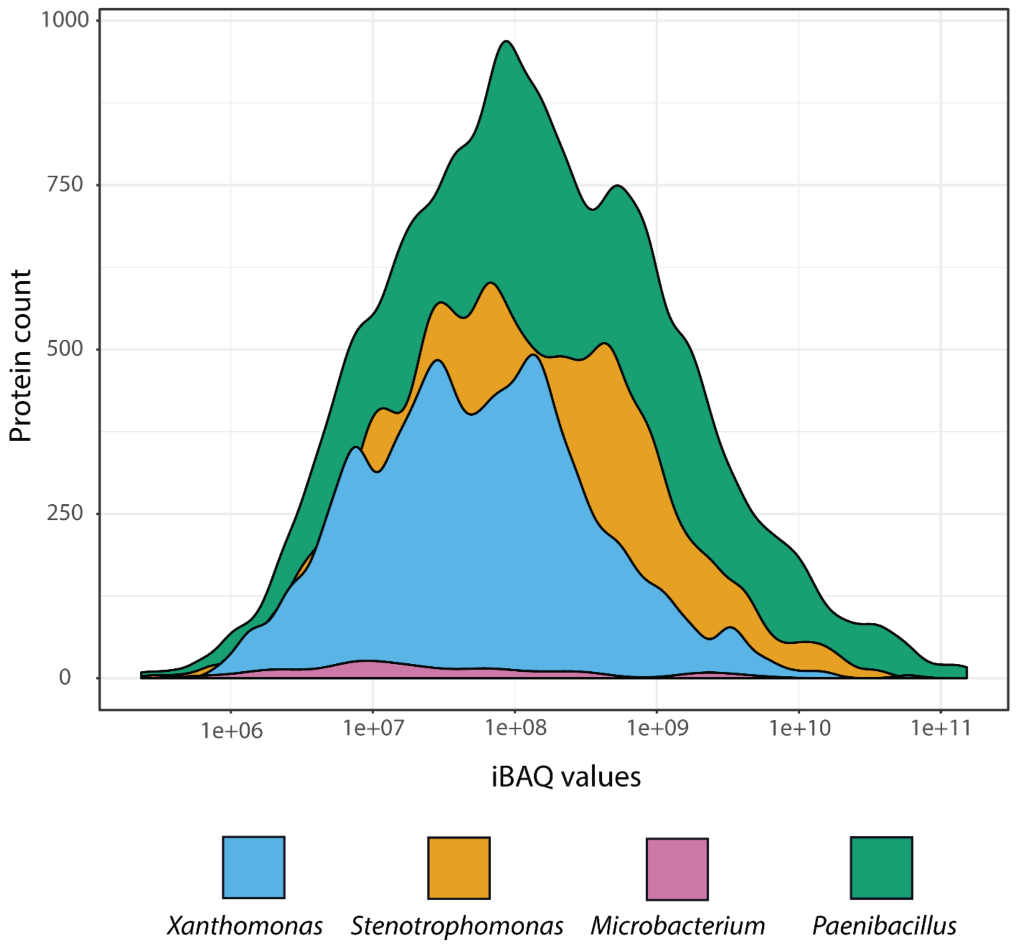

Supplementary fig. S14: Intensity based absolute quantification (iBAQ) functionality was applied to estimate the absolute label free quantification based on the summed peak intensity normalized to theoretical number of peptides in a protein[28]. Dynamic range from the least abundant to the most abundant protein spans almost 6 orders of magnitude with both the most and least abundant protein being from *Paenibacillus*. *Microbacterium* being the least abundant organism still almost spanned 4 orders of magnitude whereas *Stenotrophomonas* and *Xanthomonas* spanned approximately 5 orders of magnitude. This indicates the increased difficulty in identifying low abundant species in a multi species biofilm.

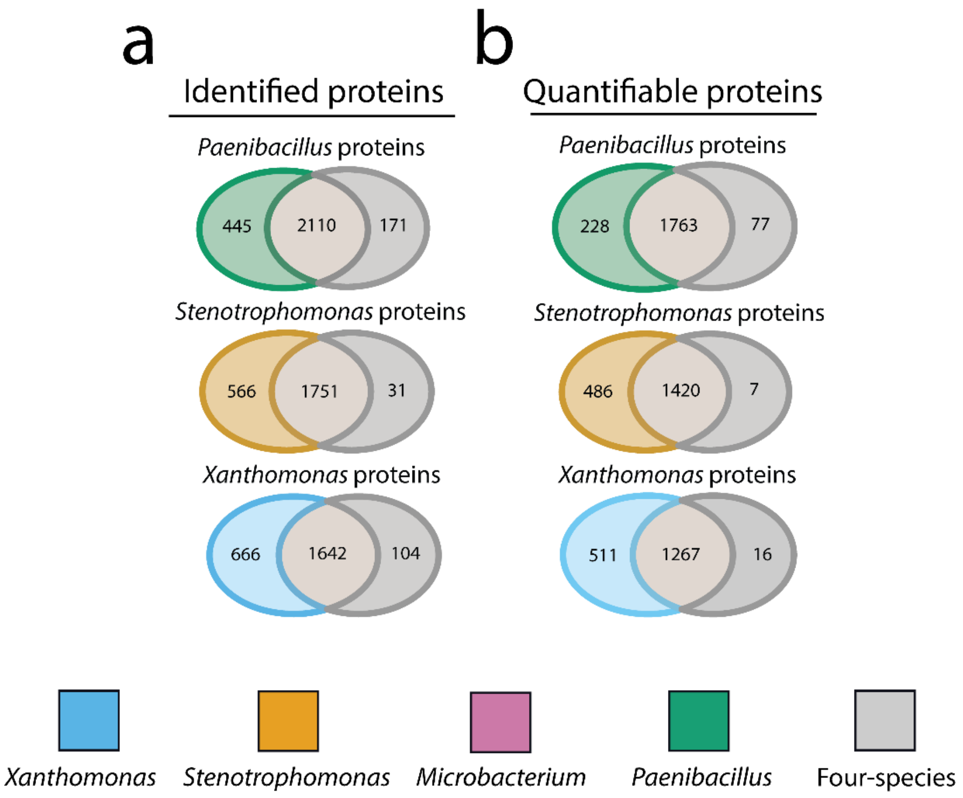

Supplementary fig. S15: Protein counts of identified and quantifiable proteins from single- and four-species samples analysed with standard reference proteomes. a) Protein counts for identified proteins, indicating overlap between single- and four-species biofilm, for each species. b) Protein counts for quantifiable proteins, indicating overlap between single- and four-species biofilm, for each species.

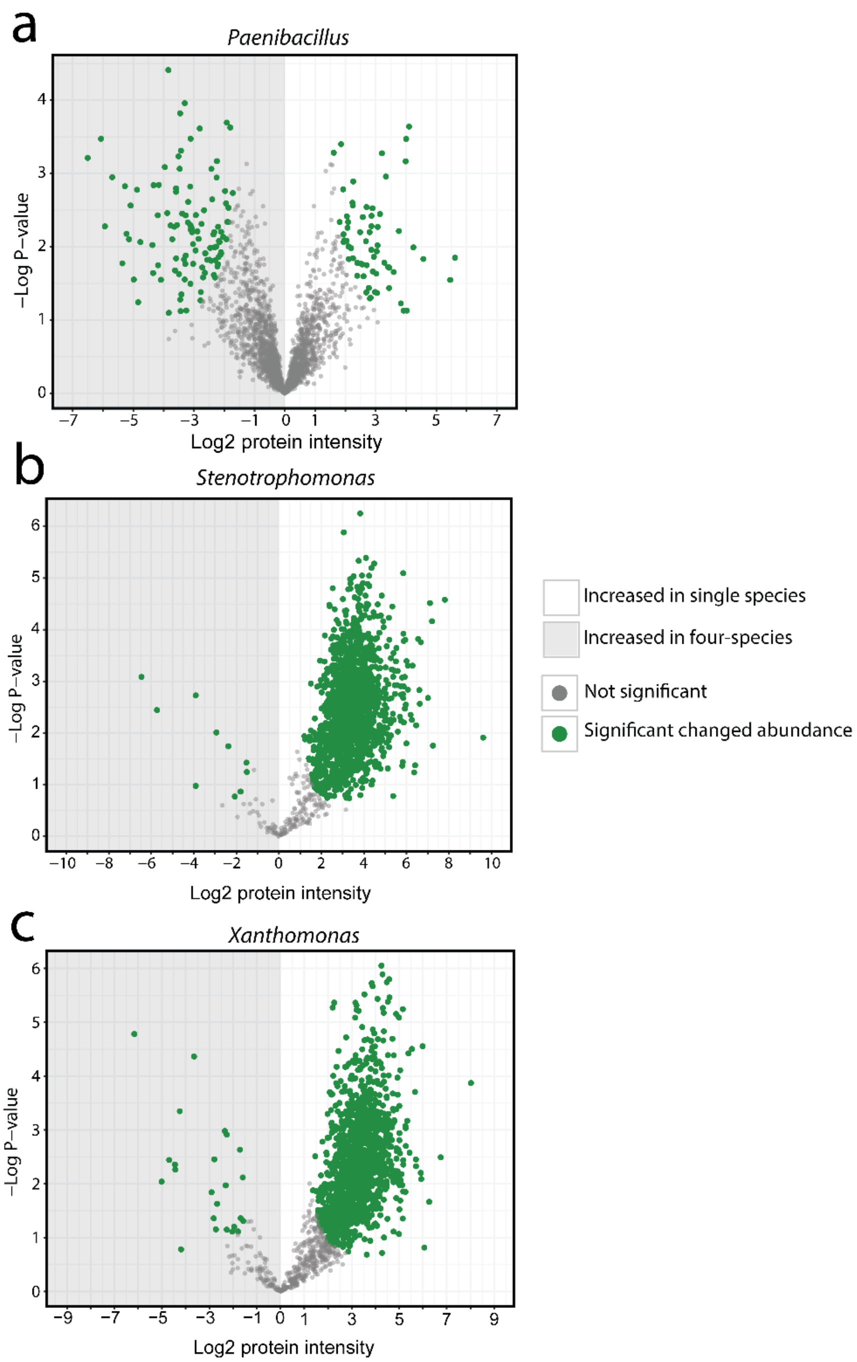

Supplementary fig. S16: Label free quantification of proteins between five biological replicates of single- and four-species biofilm identified with trimmed reference proteomes but data was analysed without MaxQuant LFQ normalisation. Proteins with significant changes in abundance were identified using a modified Welsh t-test with an S0 constant of 1 and valid values in at least 60% of both single species and four-species biofilm (three out of five replicates for both conditions). Correction for multiple hypothesis testing was performed with permutation based FDR with a significance threshold of 0.05.

Result list from Fischer’s exact test on significant *Paenibacillus* proteins

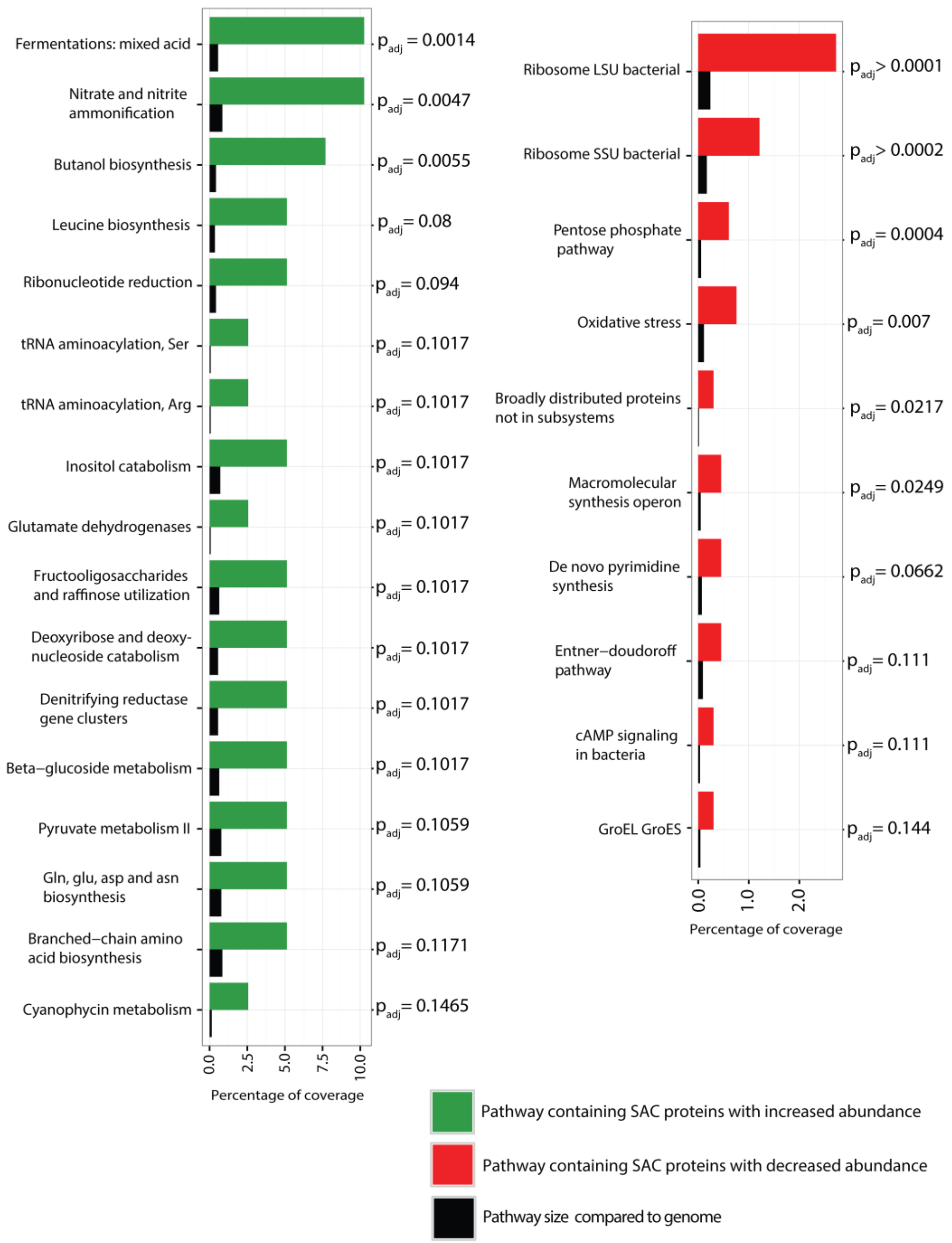

Supplementary fig. S17: Pathways with proteins significantly changed in abundance from *Paenibacillus*. Proteins with significant changes in abundance were mapped to pathways, and pathway coverage was assessed by a Fischer’s exact test. FDR adjusted p-values are presented for each pathway. Black bars display pathway size compared to the size of reference proteome, which can be grouped in pathways. Green and red color bars display the ratio of SCA proteins in the pathway compared to the total amount of SCA proteins in pathways. Pathways in red further indicate decreased abundance in the four-species biofilm, and pathways in green indicate increased protein abundance in the four-species biofilm.

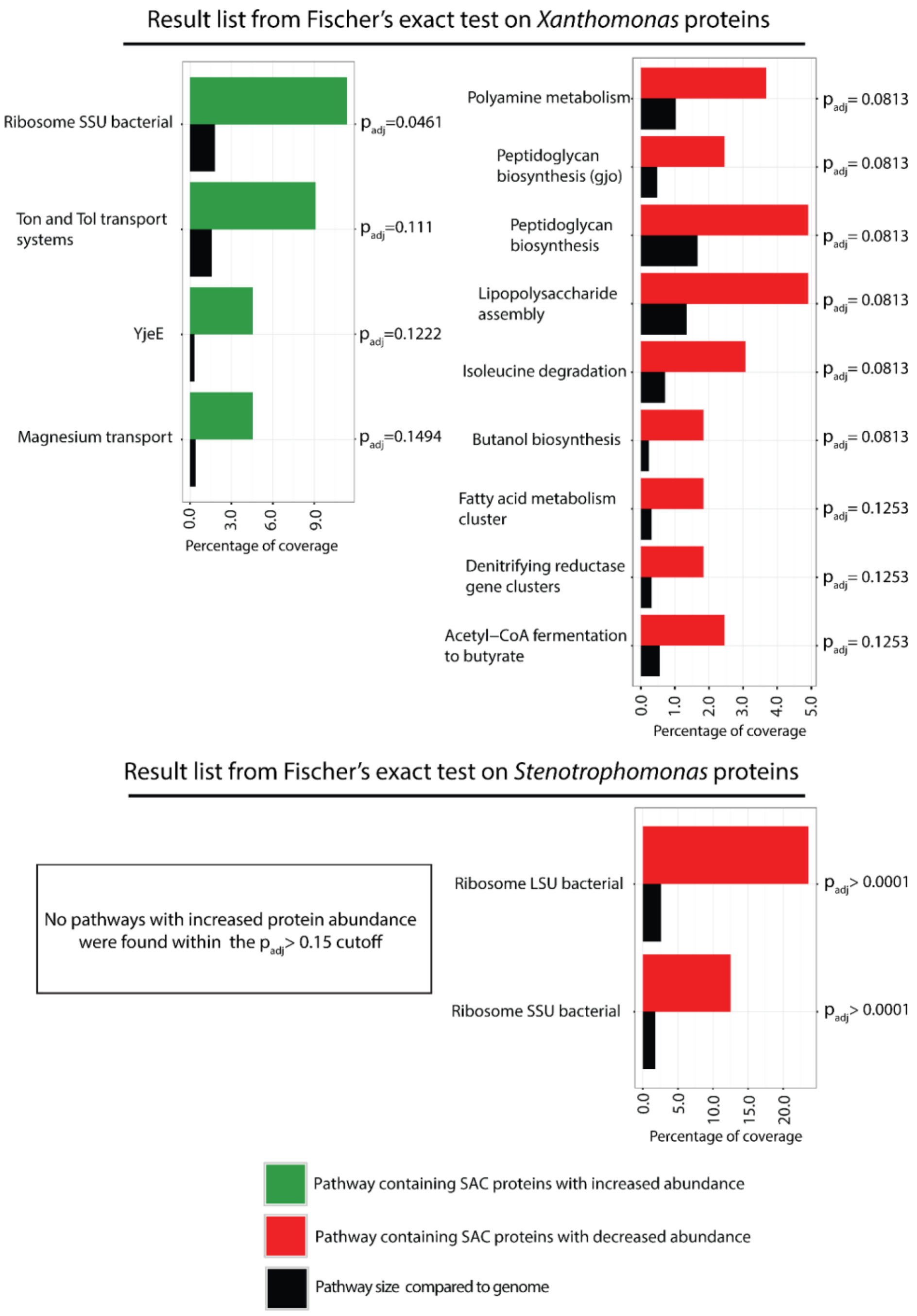

Supplementary fig. S18: Pathways with proteins significantly changed in abundance from *Xanthomonas* and *Stenotrophomonas*. Proteins with significant changes in abundance were mapped to pathways, and pathway coverage was assessed by a Fischer's exact test. Protein function and pathways were inferred using the RAST database. Black bars display pathway size compared to the size of reference proteome, which can be grouped in pathways. Green and red color bars display the ratio of SCA proteins in the pathway compared to the total amount of SCA proteins in pathways. Pathways in red further indicate decreased abundance in the four-species biofilm, and pathways in green indicate increased protein abundance in the four-species biofilm.

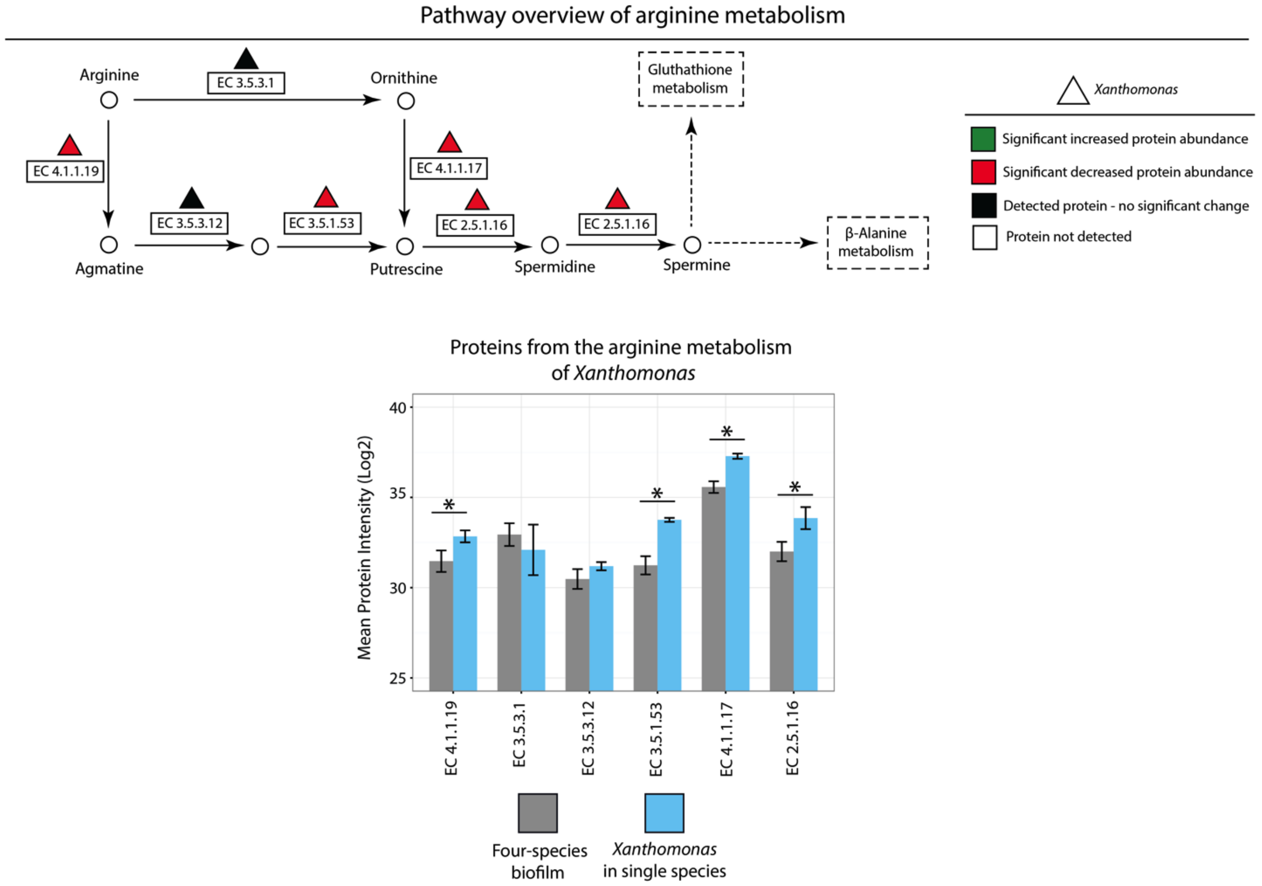

Supplementary fig. S19: Schematic overview of the arginine conversion to spermine in *Xanthomonas*, mapped with proteins changed in abundance both significantly and non-significantly. Triangles mark proteins from *Xanthomonas,* with the colour of the triangle marking the state of the protein. Green indicates a significant increase in abundance and red indicates a significant decrease in abundance in the four-species biofilm. Black indicates no change in abundance and white indicates that the protein was not quantifiable in the samples.

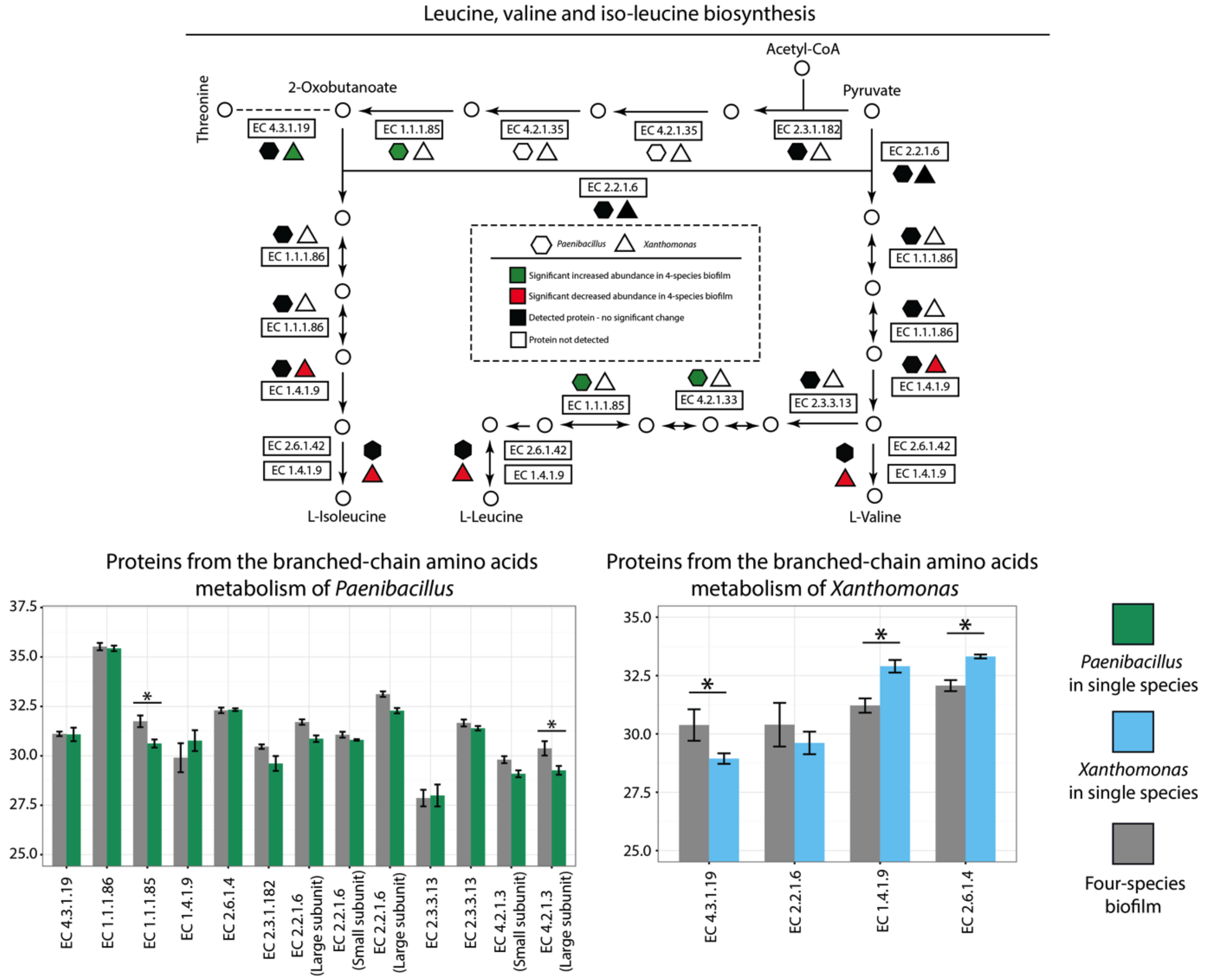

Supplementary fig. S20: Schematic overview of the leucine, valine and isoleucine biosynthesis pathway mapped with proteins changed in abundance, both significantly and non-significantly, from *Paenibacillus* and *Xanthomonas*. Hexagons and triangles mark proteins from *Paenibacillus* and *Xanthomonas*, respectively. The colour marks the state of the protein, with green indicating a significantly increased abundance and red indicating a significantly decreased abundance in the four-species biofilm. Black indicates no change in abundance and white that the protein was not quantifiable in the samples.

Supplementary tables

	Overlapping peptides from MS data			
	Xanthomonas	*Stenotrophomonas*	*Microbacterium*	*Paenibacillus*
Xanthomonas		8468	63	91
Stenotrophomonas	8468		61	84
Microbacterium	63	61		71
Paenibacillus	91	84	71	

Supplementary table S1: Identified peptides from experimental data, shared between the four species.

		Overlapping peptides from reference proteomes			
Species	*Peptides in reference proteome*	*Stenotrophomonas*	*Xanthomonas*	*Paenibacillus*	*Microbacterium*
Stenotrophomonas	62468		10956	90	68
Xanthomonas	68208	10956		104	75
Paenibacillus	102533	90	104		87
Microbacterium	56753	68	75	87	

Supplementary table S2: Shared peptides between the reference proteomes of the organisms in the four-species consortium. Total peptides in each reference proteome are indicated with a length of 7 amino acids or above. As shown, the overlap between *Xanthomonas* and *Stenotrophomonas* is much larger than the overlap between any other combinations of species.

Samples	# Bio reps	Genome size	Protein coding genes	Identified proteins	Proteome coverage (%)
Stenotrophomonas	5	4.22 Mbp	3674	2263	61.6
Xanthomonas	5	4.68 Mbp	4149	2191	52.8
Microbacterium	3	3.99 Mbp	3803	1469	38.6
Paenibacillus	5	7.27 Mbp	6430	2481	38.6
Four-species	5	-	18056	5961	33.0

Supplementary table S3: Overview of quantifiable proteins in single and four-species biofilm: Proteins found with valid values in 60% of the replicates was used for quantification. The percentage of the reference proteome used for quantification is indicated.

	% Proteins in RAST pathways			*% Hypothetical proteins*		
Species	*Reference proteome*	*Experimental proteome*	*Significant proteins (*)*	*Reference proteome*	*Experimental proteome*	*Significant proteins (*)*
Paenibacillus	*36.2*	*56.3*	*58.2*	*34.6*	*15.9*	*16.5*
Stenotrophomonas	*44.6*	*61.8*	*66.7*	*28.9*	*12.8*	*9.7*
Xanthomonas	*39.4*	*57.6*	*68.2*	*31.4*	*14.3*	*9.1*
Microbacterium	*41.2*	*NA*	*NA*	*25.7*	*NA*	*NA*
Four-species	*NA*	*61.3*	*NA*	*NA*	*12.6*	*NA*

Supplementary table S4: Overview of the coverage of proteins in reference proteomes, experimental proteomes and proteins with significant changes in abundance. In addition, the numbers of hypothetical proteins found in reference proteomes, experimental proteomes and the proteins with significant changes in abundance are indicated. (*) Proteins with significant changes in abundance are the combination of proteins from both single and four-species biofilm.

Supplementary R-script

Supplementary R-script 1: Preparation of reduced reference proteomes

```
#########################################################################################

# ------------------------------------ General information and used Packages -------------------------------------------

## updated Thursday, Sep 22, 2016 to work on any number of fasta files
## Andrea Marquard

# Install packages used in script
# source("http://bioconductor.org/biocLite.R")
# biocLite("cleaver")
# install.packages("seqinr")

# Load packages used in script
library(cleaver)
library(seqinr)
library(ggplot2)
library(reshape2)

#########################################################################################

# ---------------------------------------- Load fasta files, and do in silico digest -----------------------------------------------------------

if(interactive()) {
  files <- c("Srhizo_Wenzheng.fasta", "Xretro_JAHC.fasta", "Pamy_Wenzheng.fasta", "Moxy_Wenzheng.fasta") # write file names
here
} else {
  files <- commandArgs(TRUE)
}
## Read the Proteome fasta files.
## 'seqtype' argument describes whether it is DNA or AA.
FASTA <- lapply(files, function(f) {
  read.fasta(file = f, seqtype = "AA", as.string = TRUE)
})
# [specific to our case]
# Example:
#   from: "Moxy_fig|82380.14.peg.532"
#   to:   "Moxy_fig|82380.14.peg"
species.names <- sapply(FASTA, function(fa) {
  sub("\\.[0-9]+$", "", names(fa)[1])
})
DIGESTS <- lapply(FASTA, function(fa) {
  cleave(unlist(fa), enzym = "trypsin")
})
names(FASTA) <- names(DIGESTS) <- names(files) <- species.names

####################################################################################
#------------------------------------ Simplify the data for faster analysis --------------------------------------------------

# The list of fragments is turned into a vector, fragments shorter than 7 are
# removed and the peptides are grouped according to their length. The peptides
# need to be grouped into their peptide length so that you only compare
# peptides of eg. 11aa with peptides from the other organism that are also
# 11aa. These steps significantly reduce the time for the analysis.
preparePeptidomes <- function(x, min.length) {
  names(x) <- paste0(names(x), "_")
  x <- unlist(x)                  # Turn the list into a vector
  x <- x[nchar(x) >= min.length]    # Remove fragments shorter than 7
```

```
  x <- split(x, nchar(x))          # Group the peptides by length
  return(x)
}

###################################################################################
#-----------------------------  Find peptides that are present in more than one species  ------------------------------------------

intersectPeptidomes <- function(PLIST, ...) {
  # input must already be digested (use the output from 'cleave')
  PLIST <- lapply(PLIST, preparePeptidomes, ...)
  # Get all the combinations to compare
  pairs <- combn(length(PLIST), 2)
  # Now for each combination of proteomes...
  shared <- apply(pairs, 2, function(x) {
    P1 <- PLIST[[ x[1] ]]  # first proteome to compare
    P2 <- PLIST[[ x[2] ]]  # second proteome to compare with
    message("Intersecting proteomes ", names(PLIST)[x[1]], " and ", names(PLIST)[x[2]])
    # For each of the peptide lengths in the first proteome (7mers, 8mers, 9mers, etc...)
    tmp <- lapply(names(P1), function(l) {
      if(l %in% names(P2)) {
        # Now this function also returns the organism and protein ids,
        # so it is faster to find these peptides in the fasta files afterwards
        # Compare all peptides in 1 and 2
        mat <- outer(X = P1[[l]], Y = P2[[l]], FUN = `==`)
        # Get the organism/protein name for those that were shared
        shared_rows <- apply(mat, 1, any)
        shared_cols <- apply(mat, 2, any)
        shared_P1 <- P1[[l]][shared_rows]
        shared_P2 <- P2[[l]][shared_cols]
        # Return a vector of the shared peps
        c(shared_P1, shared_P2)
      }
      # if no overlap, returns empty char vector
      # if no peps in P2 of that length, returns NULL.
      # Doesn't really matter once you use 'unlist' on the result
    })
    unlist(tmp)
  })
  unlist(shared)
}
# Intersect all the digested proteomes
shared_peps <- intersectPeptidomes(DIGESTS, min.length = 7)

################################################################################
#--------------------------------------------   Reformat the results  ----------------------------------------------------------

# Remove the trailing number, which was introduced by R to keep track of multiple proteins from same species
# Example:
#   from: "Moxy_fig|82380.14.peg.532_18"
#   to:   "Moxy_fig|82380.14.peg.532"
prots <- sub("_[0-9]+$", "", names(shared_peps))

# Make table of protein names and peptides, one row per peptide.
sp_mat <- cbind(protein = prots, peptide = unname(shared_peps))
# Get the species name, by removing the protein id from the end
# Example:
#   from: "Moxy_fig|82380.14.peg.532"
#   to:   "Moxy_fig|82380.14.peg"
species <- sub("\\.[0-9]+$", "", prots)
# Make list of tables, one table per species
sp_list <- split.data.frame(sp_mat, species)

##############################################################################
```

```
# ------------------------------------  Remove shared peps and create new fasta files  -----------------------------------------

NEW_FASTA <- FASTA
# For each proteome/FASTA
for (i in species.names) {
  cat("\nProcessing peptides in FASTA: ", i, "\n")
  # for each line in the file...
  for (j in 1:nrow(sp_list[[i]])) {
    # current line, which consists of:
    #   1) protein (the protein name)
    #   2) peptide (the aa sequence)
    prot <- sp_list[[i]][j,]["protein"]
    pep <- sp_list[[i]][j,]["peptide"]
    # In the relevant proteome, take the relevant protein, and substitute the peptide sequence with nothing ("")
    NEW_FASTA[[i]][[prot]] <- gsub(pep, "", NEW_FASTA[[i]][[prot]])
    if(j%%1000 == 0) cat("Processed ", j, " peptides in FASTA: ", i, "\n")
  }
}

################################################################################
# -----------------------------------------------  Write new fasta files to disk  -----------------------------------------------

for (i in names(NEW_FASTA)) {
  # make new file name from the old one
  new.file <- sub("(\\.\\w+)$", ".UNIQUE\\1", files[i])
  write.fasta(NEW_FASTA[[i]],
    names = names(NEW_FASTA[[i]]),
    as.string = TRUE,
    file.out = new.file)
  message("Saved new FASTA file for species ", i, " as ", new.file)
}

########################################################################################
# --------------------------------------------  Compare peptide sets before and after   ----------------------------------------------------

# print table of non-unique peptides
write.table(do.call(rbind, lapply(names(sp_list), function(species) cbind(species, sp_list[[species]]))),
    file = "table-of-removed-peptides.tsv", quote = FALSE, sep = "\t", row.names = FALSE)
# For each proteome/FASTA
countPeps <- lapply(setNames(species.names, species.names), function(i) {
  prot_list <- split(sp_list[[i]][,"peptide"], sp_list[[i]][,"protein"])
  # for protein in that proteome...
  t(sapply(names(DIGESTS[[i]]), function(j) {
    # what's the number of digested peps in total for this protein? (only those 7 aas or longer!)
    n.total <- sum( nchar(unique(DIGESTS[[i]][[j]])) >= 7 )
    # if the protein is not in the list of redundant peptides
    if(! j %in% names(prot_list)) {
      return(c(before = n.total, Removed = 0, Retained = n.total))
    }
    # If it is, on the other hand, count the number of redundant peps in this protein
    n.removed <- length(unique(prot_list[[j]]))
    return(c(before = n.total, Removed = n.removed, Retained = n.total - n.removed))
  }))
})
countPeps <- lapply(countPeps, function(i) {
  cbind(i, "percent.removed" = i[,"Removed"] / i[,"before"])
})
for (i in names(countPeps)) {
  j <- sub("_.*", "", i)
  write.table(countPeps[[i]], file = paste0(j, "_count-peptides.tsv"), col.names = NA, quote = FALSE, sep = "\t")
}
pdf("Retained-peptides-xy.pdf", width = 5, height = 5)
for (i in names(countPeps)) {
```

```
  x <- countPeps[[i]]
  plot(x[,"before"]+1, xlab = "Peptides per protein (+1)", x[,"Retained"]+1, ylab = "Retained peptides (+1)", las = 1, main = i, log = "xy", pch  = 16, col = "#00000020")  # log scale
}
dev.off()
pdf("Fraction-peptides-removed.pdf", width = 5, height = 4)
for (i in names(countPeps)) {
  x <- countPeps[[i]]
  ord <- order(x[,"percent.removed"])
  levels <- rownames(x)[ord]
  tmp <- melt(x[,2:3])
  tmp$Var1 <- factor(tmp$Var1, levels = levels)
  tmp$Var1 <- as.numeric(tmp$Var1)
  tmp$Var2 <- factor(tmp$Var2, levels = c("Removed", "Retained"))
  colnames(tmp) <- c("Proteins", "Peptides", "Fraction")
  p <- ggplot(tmp, aes(x = Proteins, y = Fraction, fill = Peptides)) +
     geom_bar(width = 1, stat = "identity", position = "fill") +
     scale_x_continuous(expand = c(0,0)) +
     scale_y_continuous(expand = c(0,0)) +
     ggtitle(i)
  print(p)
}
dev.off()


# --------------------------------------------      DONE!!!    ----------------------------------------------------
```
